# The Clinicopathological and Prognostic Value of CCR7 Expression in Breast Cancer Throughout the Literature: A Systematic Review and Meta-Analysis

**DOI:** 10.3390/biomedicines13041007

**Published:** 2025-04-21

**Authors:** Mohamed Elhadary, Basel Elsayed, Amgad Mohamed Elshoeibi, Omar Karen, Ibrahim Elmakaty, Jehad Alhmoud, Ahmad Hamdan, Mohammed Imad Malki

**Affiliations:** 1 College of Medicine, QU Health, Qatar University, Doha P.O. Box 2713, Qatar; me1902913@qu.edu.qa (M.E.); be1905231@qu.edu.qa (B.E.); ae1902917@qu.edu.qa (A.M.E.); ok1805503@qu.edu.qa (O.K.); ah1904442@qu.edu.qa (A.H.); 2 Department of Medical Education, Hamad Medical Corporation, Doha P.O. Box 3050, Qatar; ie1703006@qu.edu.qa; 3 Department of Medical Laboratory Sciences, Jordan University of Science and Technology, Irbid 22110, Jordan; jfalhmoud@just.edu.jo; 4 Department of Basic Medical Sciences, College of Medicine, QU Health, Qatar University, Doha P.O. Box 2713, Qatar

**Keywords:** chemokine receptor 7, clinicopathologic features, meta-analysis, prognostic value, breast receptor expression, nodal metastasis

## Abstract

**Background/Objective:** This study aimed to determine the clinicopathological findings and prognostic value of chemokine receptor 7 (CCR7) expression in patients with breast cancer (BC). **Methods:** Up to the 25th of March 2025, a search was conducted using five databases: PubMed, Embase, Scopus, Medline, and Web of Science. The methodological standards for the epidemiological research scale were used to assess the quality of the included articles, and Stata software (Stata 19) was used to synthesize the meta-analysis. **Results:** We considered 12 of 853 studies that included 3119 patients with BC. High CCR7 expression was not associated with age (odds ratio [OR] 0.82, 95% confidence interval [CI] 0.66–1.03); clinicopathological findings, including tumor size (OR 1.062, 95% CI 0.630–1.791); clinical stage (OR 1.753, 95% CI 0.231–13.304); nodal metastasis (OR 1.252, 95% CI 0.571–2.741); or histological differentiation (OR 1.167, 95% CI 0.939–1.450). CCR7 expression did not affect overall survival (hazard ratio 0.996, 95% CI 0.659–1.505). **Conclusions:** Our quantitative analysis did not reveal an association between CCR7 expression and poor clinicopathological or prognostic features in BC patients. Because of the high heterogeneity and potential publication bias, large high-quality studies are required to further confirm these findings.

## 1. Introduction

Breast cancer (BC) is a multifaceted and heterogeneous disease that originates from the unregulated growth and proliferation of cells in breast tissue. It is the most frequently diagnosed cancer among women globally, with an estimated 2.3 million new cases and 685,000 fatalities reported in 2020 [1]. BC development is influenced by various factors, including genetic predisposition, age, hormonal status, lifestyle, and environmental exposures [1]. The five-year survival rate for women with localized BC is 99%, while for those with regional and distant metastatic BC, the survival rates drop to 85% and 28%, respectively [2]. Breast cancer is often classified based on whether certain molecular markers are present, such as estrogen receptors (ER), progesterone receptors (PR), and human epidermal growth factor receptor 2 (HER2). These markers have significant prognostic and therapeutic implications, as they aid in guiding treatment decisions and predicting outcomes. For example, tumors that express high levels of ER and/or PR are often susceptible to hormone therapy, while those that overexpress HER2 may benefit from targeted therapy with drugs such as trastuzumab (Herceptin) [3]. Multiple factors influence cancer treatment, including tumor size, stage, molecular subtype, and patient preferences. Common treatment modalities include surgery, radiation therapy, chemotherapy, and targeted therapy. Treatment aims to eliminate cancer or prevent its recurrence while minimizing side effects and preserving quality of life [3]. Although BC treatment has advanced in recent years, there is still a need for new targeted therapies to reduce the disease burden.

Chemokine receptor 7 (CCR7) is a G protein-coupled receptor that plays a crucial role in the migration of immune cells to lymph nodes [4]. It is expressed in various immune-cell types, including dendritic cells, T cells, and B cells. CCR7 functions by interacting with its ligands CCL19 and CCL21, which are expressed in lymph nodes and other secondary lymphoid tissues. Upon binding to its ligands, CCR7 activates intracellular signaling pathways that promote immune-cell migration towards lymph nodes [4]. In addition to its role in immune-cell migration, CCR7 has also been implicated in various pathological conditions, including cancer [4]. Studies have shown that CCR7 expression is upregulated in various types of cancer, and high CCR7 expression has been associated with poor prognosis and aggressive cancer subtypes, including non-small cell lung, gastric, and esophageal cancers. CCR7 is therefore considered a potential therapeutic target for the development of new cancer treatments. Further research is needed to fully understand the molecular mechanisms underlying CCR7 function in cancer and to determine its potential as a therapeutic target [4].

High CCR7 expression has been found to be linked to poor overall survival in breast cancer patients, independent of other clinicopathological variables [5]. Additionally, CCR7 expression is significantly correlated with advanced tumor stage and lymph-node metastasis in breast cancer patients and is associated with a poor prognosis [6]. Some studies, on the other hand, have reported contradictory findings, implying that there is no significant link between CCR7 and worsening prognosis in patients with BC [7], and one research group even suggested that CCR7 can be an indicator of better survival [8]. Moreover, the first humanized anti-CCR7 antibody was approved for the treatment of patients with relapsed/refractory chronic lymphocytic leukemia (NCT04704323), highlighting CCR7’s potential as a key target in cancer therapy. This meta-analysis aimed to determine which clinical and pathological features are linked to high levels of CCR7 expression and to assess its impact on the prognosis of BC patients.

## 2. Materials and Methods

### 2.1. Protocol and Registration

This systematic review and meta-analysis was performed using the preferred reporting items for systematic reviews and meta-analyses (PRISMA) guidelines (see the PRISMA checklist in the Supplementary Materials, Table S1) [9]. The meta-analysis protocol was submitted to the international prospective register of systematic reviews (PROSPERO) online database (PROSPERO Identifier: CRD42023403436).

### 2.2. Search Strategy

On November 20th, 2022, we developed our search strategy using the PubMed database, employing two medical subject headings (MeSH) terms, “Breast Neoplasms” [MeSH] AND “Receptors, CCR7” [MeSH], along with non-MeSH keywords centered on those terms to avoid missing related articles. There were no language or time constraints in the search. Using a polyglot translator [10], the developed search strategy was transferred to Embase, Scopus, Medline (EBSCO), and Web of Science. The search strategy was updated on 29 March 2025, to capture any newly published articles on the topic. The entire strategy for accessing each database is included in the Supplementary Materials. The studies were then transferred to EndNote X7, where duplicates were detected and eliminated.

### 2.3. Eligibility Criteria

Our meta-analysis included studies that (1) focused on the detection of CCR7 in neoplastic cells in patients with pathologically identified BC; (2) measured CCR7 expression; (3) used immunohistochemistry; (4) assessed CCR7 expression in primary BC tissues; (5) used negative controls for comparison; and (6) revealed a correlation between CCR7 expression and any of the clinicopathological features and prognostic indicators, including tumor staging, tumor size, lymph-node metastasis, recurrence, and overall survival (OS), and used an observational study design. We excluded research articles from our meta-analysis if they were (1) reviews or nonoriginal articles, (2) based on animal BC samples, or (3) duplicate or similar across studies. Only articles with more details or the most recent article were included if more than one study used the same BC data.

### 2.4. Study Selection and Screening

After applying our search strategy to the databases we selected, the remaining articles were uploaded to the Rayyan platform for screening [11], and duplicates were removed using Endnote. Two reviewers separately reviewed the titles and abstracts, and any differences were resolved by consensus. The full texts of the eligible papers were then obtained and separately double-screened by two reviewers, with any differences resolved through team discussion.

### 2.5. Data Extraction

The extracted data included the publication year, country, study design, type of cancer, sample size, patient demographics (age and gender), number of individuals with elevated CCR7 expression, cancer stage, presence of metastasis, detection techniques, and clinicopathological features such as ER, PR, and HER-2 expression. Based on the reporting of each article, CCR7 expression was categorized as high/low or present/absent. The OS hazard ratio (HR) and its 95% confidence interval (CI) were obtained as reported in the original articles. Two investigators independently examined and obtained data from the eligible studies. When they were unable to reach an agreement, they held a meeting with all team members. These studies were included in the meta-analysis if all team members agreed.

### 2.6. Quality of Studies

To evaluate the quality of the studies included in our bias-adjusted synthesis, we used the methodological standards for epidemiological research (MASTER) scale, which employs unified approaches to assess methodological quality across various analytic research designs [12]. The MASTER scale consists of 36 safeguard questions, each requiring a binary response, with one point assigned if the safeguard was implemented and no points if it was not. These 36 safeguards are divided into seven categories: equal recruitment, equal retention, equal ascertainment, equal implementation, equal prognosis, adequate analysis, and temporal precedence [12]. Two investigators independently assessed the quality of the included studies using this tool, with a third reviewer consulted to resolve any disagreements.

### 2.7. Outcomes

The goal of this meta-analysis was to assess the clinicopathological features of different levels of CCR7 in BC patients and to determine whether CCR7 expression has any prognostic value in BC patients. Age, tumor size, clinical stage, lymph-node metastasis status, histologic differentiation status, ER receptor expression, PR receptor expression, and HER-2 receptor expression were evaluated as estimators of clinicopathological outcomes. OS was the effect size used to quantify the prognostic value of CCR7. Because only two datasets were available, recurrence and disease-free survival were not considered as outcomes. To assess OS, the hazard ratio (HR) and 95% confidence interval (CI) were calculated. A two-group comparison of binary outcomes was used to calculate the estimated odds ratios (ORs) based on the number of events and nonevents in the high and low CCR7 expression groups for the meta-analysis of age, tumor size, clinical stage, lymph-node metastasis, histologic differentiation, ER receptor expression, PR receptor expression, and HER-2 receptor expression.

### 2.8. Data Analysis

This meta-synthesis analysis was similar to that published by our group on head and neck cancer [13]. To synthesize outcome estimates and their confidence limits, the study used the quality-effect model to adjust for methodological quality-related variability [14,15]. The model disperses the study weights by quality rank, adjusting the synthesis. The outcomes of the quality assessment were represented as relative rankings [16]. The pooled-estimate analysis results are shown using forest plots. A post hoc continuity correction adding (0.5) to all cells with nonevents was used to calculate the OR. The I-squared (I^2^) statistic and the Cochran Q test *p* value were employed to assess the presence of heterogeneity [17]. Publication bias and potential small-study effects were analyzed using Doi plots [18], with the LFK index applied to measure the symmetry of the Doi plots. Heterogeneity was considered significant if the Cochran Q test yielded a *p* value below 0.05 or if the I^2^ exceeded 50%. Standard funnel plots and Egger’s regression test *p* value were also calculate to confirm the findings from the Doi plots and LFK index [19]. Moreover, a leave-one-out sensitivity analysis was performed by systematically excluding individual studies to observe any shifts in pooled estimates or heterogeneity, to identify particular studies contributing to heterogeneity. The “metan” and “lfk” modules were installed [18,20], and Stata version 16 (College Station, TX, USA) was used for all analyses, graphs, and plots. One study did not provide the HR for overall survival, so WebPlotDigitizer (Version 4) was used to extrapolate the curve, and the ipdfc module in Stata was used to generate the HR and its confidence limits [21,22].

## 3. Results

### 3.1. Study Selection

**Figure 1:**
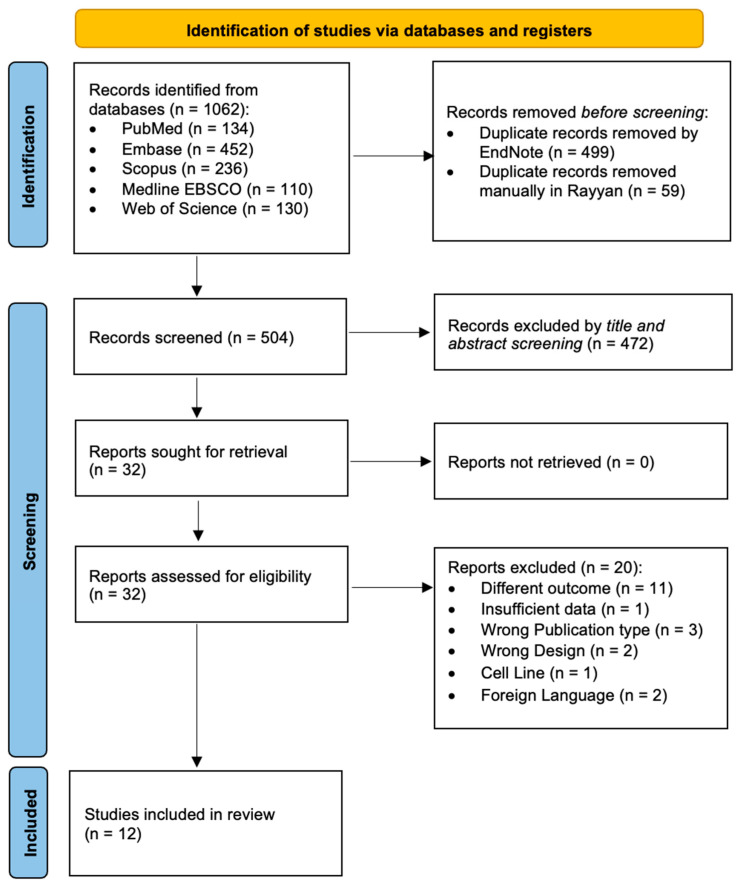
PRISMA flow chart for the systematic review and meta-analysis.

Figure 1 presents a PRISMA flow diagram illustrating the stages of study selection for this meta-analysis. The database search initially identified 1062 potential studies. After using EndNote and Rayyan to eliminate 558 duplicates, 504 unique articles were subjected to a preliminary review of their titles and abstracts. This initial screening phase led to the exclusion of 472 studies, narrowing the selection to 32 articles for a full-text review. During the detailed examination of these 32 studies, 20 were excluded for the reasons outlined in Figure 1. Consequently, 12 studies were deemed eligible and included in the meta-analysis. A comprehensive summary of the excluded studies and the specific reasons for their exclusion can be found in Table S2.

### 3.2. Study Characteristics and Data Collection

Table 1 displays the characteristics and data collected from the included studies. In summary, these articles were published from 2005 to 2021, with the majority (n = 4) originating from China and the others from countries such as France, Taiwan, the United Kingdom, the United States, Iran, and Brazil. Of the 12 studies included, two focused on triple-negative breast cancer, two on invasive ductal carcinoma, one on inflammatory breast cancer, one on HER2-amplified breast cancer, one on axillary node-positive breast cancer, and the remaining five on breast cancer as a whole. The sample sizes for these studies ranged from 44 to 1096 patients, with a median age of 49 to 51 years. Studies that assessed the correlation between CCR7 expression and age reported a cut-off of 50 years. For the correlation between CCR7 expression and tumor size, one study reported a cut-off diameter of 3 cm, while the other studies (n = 11) reported a cut-off diameter of 2 cm. Two studies that assessed clinical staging reported only distant metastasis as M0 versus M1, one study enrolled only stage III patients, and the remaining studies (n = 4) divided patients into stages I, II, III, and IV or stages I and II versus stages III and IV. For nodal metastasis, two studies used axillary nodal stages N0 and N1 versus N2 and N3, one study enrolled only patients with nodal metastasis, and the remaining studies (n = 5) reported nodal metastasis versus no nodal metastasis. All variations are highlighted in Table 1. The expression of CCR7 in breast cancer patients was evaluated with respect to age (n = 5), tumor size (n = 5), clinical stage (n = 6), nodal metastasis (n = 7), histological differentiation (n = 7), estrogen receptor expression (n = 6), progesterone receptor expression (n = 4), HER2/neu expression (n = 6), and overall survival (n = 8).

### 3.3. Quality Assessment

**Figure 2:**
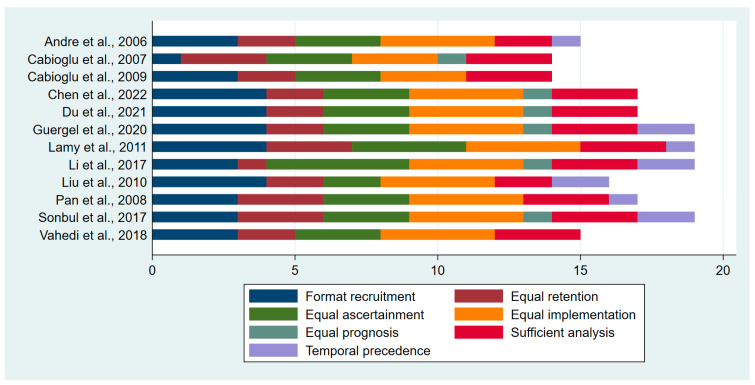
Quality scores for the included studies.

The studies included in the analysis had varying levels of safeguards, with the number of safeguards ranging from 14 to 19 out of a total of 36 and an average of 17 safeguards per study across the seven domains of the MASTER scale previously mentioned. Most of the studies demonstrated a good implementation of recruitment, retention, and ascertainment and a sufficient analysis of safeguards, with the primary differences being in the format recruitment and temporal precedence safeguards. Figure 2 displays the specific safeguards used in each study, and Table S3 in the Supplementary Materials presents the complete responses to all quality-assessment questions.

### 3.4. CCR7 Expression and Age

**Figure 3:**
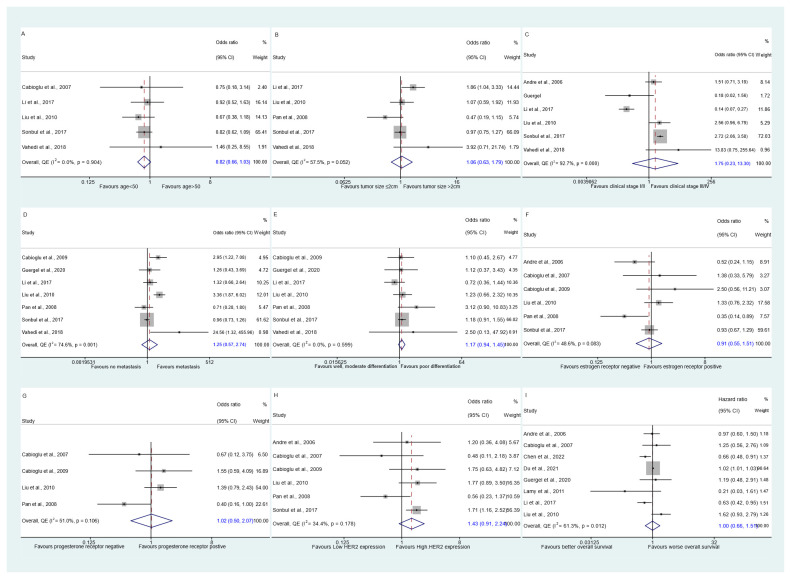
Forest plots of the meta-analysis summarizing our pooled estimates: (**A**) odds ratio for age as an outcome; (**B**) odds ratio for tumor size as an outcome; (**C**) odds ratio for clinical stage as an outcome; (**D**) odds ratio for nodal metastasis as an outcome; (**E**) odds ratio for histological differentiation as an outcome; (**F**) odds ratio for estrogen receptor expression as an outcome; (**G**) odds ratio for progesterone receptor expression as an outcome; (**H**) odds ratio for HER2/neu expression as an outcome; (**I**) hazard ratio for overall survival.

Table 2 provides an overview of the primary findings from this meta-analysis examining the association between CCR7 expression and the outcomes of interest. The analysis of CCR7 expression in relation to age included data from five studies encompassing a total of 1368 patients. The results showed no statistically significant association, with an odds ratio (OR) of 0.82 (95% CI: 0.66 to 1.03), no observed heterogeneity (I^2^ = 0.0%), and an Egger’s test *p* value of 0.676. The funnel plot, displayed in Figure S1, along with the Doi plot (Figure S2), revealed a minor positive asymmetry, indicated by an LFK index of 1.65. This asymmetry suggests a publication bias, where smaller studies tended to report more significant results (Figure 3A).

### 3.5. CCR7 Expression and Clinicopathological Features

**Figure 4:**
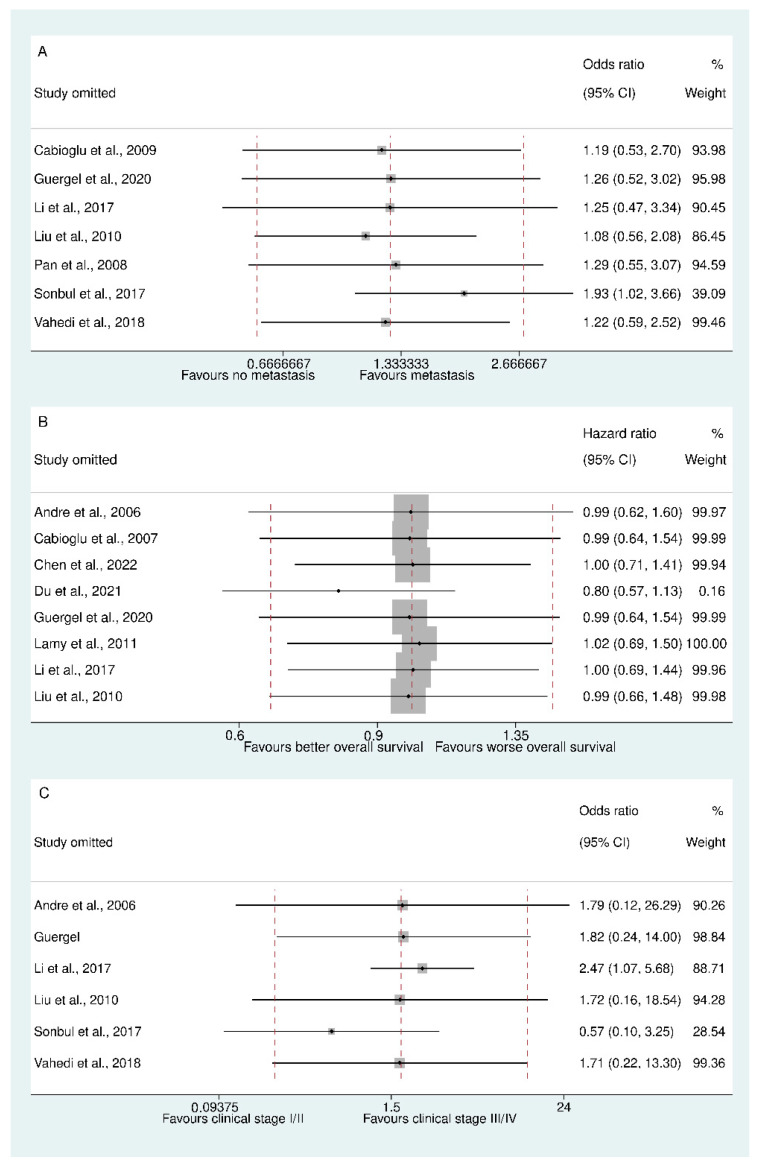
Sensitivity analysis: (**A**) influence of clinical stage; (**B**) influence of nodal metastasis; (**C**) influence of overall survival.

Analysis of CCR7 expression in relation to the clinicopathological features of breast cancer patients did not reveal any statistically significant associations. Across five studies with a total of 1403 subjects, high CCR7 levels were not significantly associated with tumor size (OR 1.062, 95% CI 0.630 to 1.791; Egger’s test *p* value 0.621; LFK index 2.59 [major asymmetry]; Figure 3B) (see the funnel and Doi plots in Figures S3 and S4). There was significant heterogeneity in the outcome of tumor size (I-squared = 57.5%), but further analysis revealed that no single study was responsible for this heterogeneity (as shown in Figure S19). Similarly, six studies with a total of 1529 subjects showed no significant association between high CCR7 levels and clinical stage (OR 1.753, 95% CI 0.231 to 13.304; Egger’s test *p* value 0.465; LFK index −2.38 [major asymmetry]; Figure 3C) (see the funnel and Doi plots in Figures S5 and S6). Likewise, the outcome of clinical stage demonstrated exceedingly high heterogeneity (I-squared = 92.7%). However, leave-one-out analysis revealed that Sonbul et al.’s study was responsible for a considerable amount of heterogeneity (as shown in Figure 4A) [7]. Furthermore, seven studies with a total of 1658 subjects found no significant association between high CCR7 levels and nodal metastasis (OR 1.252, 95% CI 0.571 to 2.741; Egger’s test *p* value 0.184; LFK index 4.25 [major asymmetry]; Figure 3D) (see the funnel and Doi plots in Figures S7 and S8). Likewise, the outcome of nodal metastasis demonstrated considerably high heterogeneity (I-squared = 74.6%), which was once again partially explained by Sonbul et al.’s study [7], as revealed by leave-one-out analysis (as shown in Figure 4B). High CCR7 expression was also not significantly associated with histological differentiation in seven studies with a total of 1646 subjects (OR 1.167, 95% CI 0.939 to 1.450; I-squared 0.0%; Egger’s test *p* value 0.601; LFK index 1.63 [minor asymmetry]; Figure 3E) (see the funnel and Doi plots in Figures S9 and S10).

### 3.6. CCR7 Expression and Breast Cancer Biomarkers

High CCR7 expression did not show a significant association with estrogen receptor status across six studies with a total of 1380 subjects (OR 0.913, 95% CI 0.553 to 1.507; I-squared 48.6%; Egger’s test *p* value 0.957; LFK index 0.93 [no asymmetry]; Figure 3F) (see the funnel and Doi plots in Figures S11 and S12), progesterone receptor status across four studies with a total of 502 subjects (OR 1.018, 95% CI 0.500 to 2.070; I-squared 51.0%; Egger’s test *p* value 0.535; LFK index −2.59 [major asymmetry]; Figure 3G) (see the funnel and Doi plots in Figures S13 and S14), or HER2/neu status across six studies with a total of 1486 subjects (OR 1.425, 95% CI 0.906 to 2.242; I-squared 34.4%; Egger’s test *p* value 0.172; LFK index −3.94 [major asymmetry]; Figure 3H) (see the funnel and Doi plots in Figures S15 and S16). Both estrogen and progesterone receptor biomarkers demonstrated mild heterogeneity (I-squared = 48.6% and I-squared = 51.0%, respectively). However, in the leave-one-out analysis, no single study was identified (as shown in Figures S20 and S21).

### 3.7. CCR7 Expression and Overall Survival

Overall survival was evaluated in eight studies involving a total sample size of 1917 subjects, which revealed a pooled HR of 0.996 (95% CI 0.659 to 1.505; Egger’s test *p* value 0.351; Figure S17), suggesting that there was no statistically significant association between CCR7 expression and overall survival. The Doi plot showed a major negative asymmetry (LFK index = −3.24, Figure S18), suggesting that studies with higher HRs had a greater likelihood of being published (Figure 3I). Moreover, the overall survival outcome demonstrated moderate heterogeneity (I-squared = 61.3%). Nevertheless, a subsequent analysis revealed that extrapolated HRs did not introduce significant heterogeneity and were congruent with the reported HRs (as shown in Figure 4C).

## 4. Discussion

To the best of our knowledge, this is the first meta-analysis assessing the associations between CCR7 expression and clinicopathological features and OS in BC patients. CCR7 was selected as the focus of this study due to its previously reported associations with poor overall survival, advanced tumor stage, and lymph-node metastasis in breast cancer patients, as highlighted in the introduction. These findings suggest a potential role for CCR7 in breast cancer progression and prognosis. However, contradictory results in the literature, including studies showing no significant association or even a potential link to better survival, have created uncertainty about its role. In this meta-analysis, a total of 12 studies exploring the association between CCR7 expression and the clinicopathological features of breast cancer patients were evaluated. The studies included in the analysis had varying levels of safeguards, with an average of 17 safeguards per study. We found no association between CCR7 expression and any of the clinicopathological features of BC (tumor size, clinical stage, nodal metastasis, histological differentiation, ER, PR, HER2/neu expression). The pooled estimate of eight studies evaluating the association between CCR7 expression and overall survival was also nonsignificant (HR 0.996, 95% CI 0.659–1.505).

The analysis revealed no significant associations between CCR7 expression and tumor size, except for the study conducted by Li et al. [8]. Nonetheless, this study used a different cut-off value of 3 cm for tumor size classification, whereas other studies included in the analysis used a 2 cm cut-off value. This discrepancy may account for the observed deviation of the results of the study from the pooled estimate. Furthermore, our findings indicated no significant association between CCR7 expression and clinical stage based on the pooled estimate. However, the study conducted by Sonbul et al. showed a significant positive association between these variables [7]. Further analysis using the leave-one-out method revealed that this study contributed significantly to the heterogeneity observed in the association. This may be attributed to the fact that the study analyzed a sample of breast cancer patients without specifying the subtypes included in the sample. Additionally, the authors reported distant metastasis instead of clinical stages I, II, III, and IV for the tumors, which could have influenced the results.

Pooled-estimate analysis revealed no significant association between CCR7 expression and nodal metastasis. However, the studies conducted by Cabioglu et al., Liu et al., and Vahedi et al. showed a significant positive association [24,30,32]. The observed differences could be attributed to the fact that Cabioglu et al.’s study included only inflammatory breast cancer patients, while the other two studies included patients with invasive ductal carcinoma [24]. These differences in breast cancer subtypes from the other studies included in the analysis may have influenced the association, thus accounting for the observed heterogeneity in the results.

Several papers have highlighted the role of CCR7 in the progression and metastasis of colorectal [33], gastric [34], esophageal [35], and prostate cancers [36]. A meta-analysis of 30 studies investigating the role of CCR7 in solid tumors, including BC, revealed that a higher expression of CCR7 was associated with poorer overall survival (HR 1.79, 95% CI 1.49–2.16, *p* < 0.001) and progression-free survival (HR 2.18, 95% CI 1.49–3.18, *p* < 0.001). However, subgroup analysis by tumor type revealed no significant association between CCR7 expression and OS in BC studies (HR 1.24, 95% CI 0.94–1.62; *p* = 0.240) [37]. Notably, only three studies on BC were included. Another meta-analysis of four studies examining the role of CCR7 expression in OS in patients with esophageal squamous cell carcinomas revealed that high CCR7 expression was correlated with a poorer prognosis (HR 2.06, 95% CI 1.56–2.71, *p* < 0.001) [38]. Du et al. conducted a meta-analysis of 15 studies on CCR7 expression and clinicopathological findings in gastric cancer. They found that CCR7 expression was associated with deeper tumor invasion, advanced stage, vascular invasion, and lymph-node metastasis [39]. However, we could not find such an association in our study.

Our meta-analysis has several limitations that must be acknowledged. First, the included studies showed variation in methodologies, including CCR7 expression categorization, tumor size cut-offs, and clinical staging criteria, which may contribute to inconsistencies and reduce the generalizability of our findings. The lack of detailed subtype information further limited our ability to perform subgroup analyses, restricting insights into CCR7’s role across different breast cancer contexts. In addition, five out of the included studies were published before 2010. This could suggest that the analysis was influenced by the significant changes in technology for screening and methodological analysis. Non-standardized screening and diagnostic protocols among the included studies introduced variability in patient populations and disease stages, which may have influenced the associations observed. Publication bias and small study effects were also evident, with smaller studies favoring significant results, potentially inflating effect sizes. Finally, certain analyses were based on small sample sizes, restricting statistical power. Despite these limitations, our study population was diverse, including individuals from seven countries. Nonetheless, large, high-quality, prospective studies are essential to confirm these findings, address heterogeneity, and better evaluate CCR7 expression’s role in breast cancer.

## 5. Conclusions

Our meta-analysis examined the relationship between CCR7 expression and various outcomes of interest in breast cancer patients. The analysis did not reveal any significant associations between high CCR7 expression and clinicopathological features, including age, tumor size, clinical stage, nodal metastasis, histological differentiation, estrogen receptor status, progesterone receptor status, or HER2/neu status. Furthermore, there was no statistically significant association between CCR7 expression and overall survival. However, smaller studies favored publishing more significant results, and studies with higher hazard ratios had a greater likelihood of being published. Further longitudinal studies with larger sample sizes are needed to validate our results.

## Figures and Tables

**Table 1:** The characteristics of the included studies and extracted data.

Study Characteristics			Tumor Characteristics			Age		Tumor Size		Clinical Stage		Nodal Metastasis		Histological Differentiation		Estrogen Receptor		Progesterone Receptor		HER2/Neu Receptor		Overall Survival		
Study	Study Design	Country	Tumor Type	Median Age	CCR7	>50	<50	>2 cm	<=2 cm	III, IV	I, II	Yes	No	Poor	Well, Moderate	Low	High	Low	High	Low	High	HR	Lower Limit	Upper Limit
(Andre et al., 2006) [23]	retrospective cohort	France	Axillary node positive BC	49	High					35	58	93	0			44	39			83	10	0.97 *	0.6	1.5
					Low					14	35	44	0			28	13			40	4			
(Cabioglu et al., 2007) [24]	retrospective cohort	USA	Inflammatory BC		High	4	6			10	0					5	5	2	8	7	3	1.25	0.56	2.76
					Low	16	18			34	0					13	18	9	24	18	16			
(Cabioglu et al., 2005) [25]	retrospective cohort	USA	BC		High							20	8	8	20	25	2	21	6	20	6			
					Low							73	86	42	115	130	26	106	47	134	23			
(Chen et al., 2020) [26]	retrospective cohort	China	BC		High																	0.66	0.479	0.908
					Low																			
(Du et al., 2021) [27]	retrospective cohort	China	BC		High																	1.023 *	1.015	1.031
					Low																			
(Gurgel et al., 2020) [28]	Retrospective cohort	Brazil	Triple-negative BC		High					1	31 ^b^	9	23 ^c^	14	7							1.19	0.48	2.91
					Low					6	33	9	29	25	14									
(Lamy et al., 2011) [29]	Prospective cohort	France	HER2-amplified BC		High																	0.21	0.03	1.61
					Low																			
(Li et al., 2017) [8]	Retrospective cohort	China	Triple-negative BC		High	42	44	52	34 ^a^	14	72	69	17	17	69							0.633 *	0.422	0.951
					Low	52	50	46	56	60	42	77	25	26	76									
(Liu et al., 2010) [30]	Retrospective cohort	China	IDC	51	High	54	57	74	37	17	92	70	41	32	79	59	52	59	52	80	31	1.62	0.93	2.79
					Low	52	37	58	31	6	83	30	59	22	67	41	48	40	49	73	16			
(Pan et al., 2008) [31]	Retrospective cohort	Taiwan	BC		High			15	26			13	28	11	30	19	22	19	22	21	20			
					Low			21	17			15	23	4	34	27	11	26	12	14	24			
(Sonbul et al., 2017) [7]	Retrospective cohort	England	BC		High	257	153	213	197	253	151 ^b^	151	255 ^c^	214	192	252	96			324	71			
					Low	306	150	240	216	174	282	175	283	222	236	299	106			397	51			
(Vahedi et al., 2018) [32]	Case-control	Iran	IDC		High	27	37	51	13	33	31	42	22	10	54									
					Low	2	4	3	3	0	6	0	6	0	6									

^a^ the diameter cut-off was 3 cm. ^b^ divided into M0 vs. M1. ^c^ divided into N0/1 vs. N2/3. * Multivariate survival analysis. Abbreviations: CCR7, chemokine receptor 7; HR, hazard ratio; BC, breast cancer; IDC, invasive ductal carcinoma; USA, United States of America.

**Table 2:** The key findings of the meta-analyses of CCR7 expression and outcomes of interest.

Outcome	Study No.	Sample Size	Effect Size	Estimate	95% CI	Z Score, *p* Value	I-Squared	Egger’s *p* Value
Age	5	1368	Odds ratio	0.822	0.656, 1.029	−1.709, 0.087	0.0	0.676
Tumor size	5	1403	Odds ratio	1.062	0.630, 1.791	0.227, 0.820	57.5	0.621
Clinical stage	6	1529	Odds ratio	1.753	0.231, 13.304	0.543, 0.587	92.7	0.465
Nodal metastasis	7	1658	Odds ratio	1.252	0.571, 2.741	0.561, 0.575	74.6	0.184
Histological differentiation	7	1646	Odds ratio	1.167	0.939, 1.450	1.392, 0.164	0.0	0.601
Estrogen receptor	6	1380	Odds ratio	0.913	0.553, 1.507	−0.356, 0.722	48.6	0.957
Progesterone receptor	4	502	Odds ratio	1.018	0.500, 2.070	0.049, 0.961	51.0	0.535
HER2/neu	6	1486	Odds ratio	1.425	0.906, 2.242	1.532, 0.126	34.4	0.172
Overall survival	8	1917	Hazard ratio	0.996	0.659, 1.505	−0.020, 0.984	61.3	0.351

Abbreviations: CI, confidence interval; HER2, human epidermal growth factor receptor 2.

## Data Availability

The original contributions presented in this study are included in the article/supplementary material. Further inquiries can be directed to the corresponding author.

## Supplementary Materials

The following supporting information can be downloaded at: https://www.mdpi.com/article/10.3390/biomedicines13041007/s1, Table S1: Prisma checklist; Table S2: Excluded articles at full-text screening; Table S3: Quality-assessment scores; Figure S1: Funnel plot for age publication bias; Figure S2: Doi plot for age publication bias; Figure S3: Funnel plot for tumor size publication bias; Figure S4: Doi plot for tumor size publication bias; Figure S5: Funnel plot for clinical stage publication bias; Figure S6: Doi plot for clinical stage publication bias; Figure S7: Funnel plot for nodal metastasis publication bias; Figure S8: Doi plot for nodal metastasis publication bias; Figure S9: Funnel plot for histological differentiation publication bias; Figure S10: Doi plot for histological differentiation publication bias; Figure S11: Funnel plot for estrogen receptor publication bias; Figure S12: Doi plot for estrogen receptor publication bias; Figure S13: Funnel plot for progesterone receptor publication bias; Figure S14: Doi plot for progesterone receptor publication bias; Figure S15: Funnel plot for HER2/neu publication bias; Figure S16: Doi plot for HER2/neu publication bias; Figure S17: Funnel plot for overall survival publication bias; Figure S18: Doi plot for overall survival publication bias; Figure S19: Leave-one-out analysis for tumor size; Figure S20: Leave-one-out analysis for estrogen receptor; Figure S21: Leave-one-out analysis for progesterone receptor.
