# Supplementary material for: The Clinicopathological and Prognostic Value of CCR7 Expression in Breast Cancer Throughout the Literature: A Systematic Review and Meta-Analysis
Source: Biomedicines. 2025 Apr 21;13(4):1007. doi: 10.3390/biomedicines13041007 (PMC12024592; doi:10.3390/biomedicines13041007)
Supplement: Supplementary file 1 [file biomedicines-13-01007-s001.zip › biomedicines-3606613-supplementary.pdf]

Supplementary Material

The Clinicopathological and Prognostic Value of CCR7 Expression in Breast Cancer Throughout the Literature: A Systematic Review and Meta-Analysis

Mohamed Elhadary [1], Basel Elsayed [1], Amgad Mohamed Elshoeibi [1], Omar Karen [1], Ibrahim Elmakaty [2], Jehad Alhmoud [3], Ahmad Hamdan [1] and Mohammed Imad Malki [4,*]

Table S1: Prisma checklist

Section and Topic	Item #	Checklist item	Location where item is reported
**TITLE**			
Title	1	Identify the report as a systematic review.	1
**ABSTRACT**			
Abstract	2	See the PRISMA 2020 for Abstracts checklist.	1
**INTRODUCTION**			
Rationale	3	Describe the rationale for the review in the context of existing knowledge.	1
Objectives	4	Provide an explicit statement of the objective(s) or question(s) the review addresses.	3
**METHODS**			
Eligibility criteria	5	Specify the inclusion and exclusion criteria for the review and how studies were grouped for the syntheses.	4
Information sources	6	Specify all databases, registers, websites, organisations, reference lists and other sources searched or consulted to identify studies. Specify the date when each source was last searched or consulted.	4
Search strategy	7	Present the full search strategies for all databases, registers and websites, including any filters and limits used.	Supplementary material
Selection process	8	Specify the methods used to decide whether a study met the inclusion criteria of the review, including how many reviewers screened each record and each report retrieved, whether they worked independently, and if applicable, details of automation tools used in the process.	4
Data collection process	9	Specify the methods used to collect data from reports, including how many reviewers collected data from each report, whether they worked independently, any processes for obtaining or confirming data from study investigators, and if applicable, details of automation tools used in the process.	5
Data items	10a	List and define all outcomes for which data were sought. Specify whether all results that were compatible with each outcome domain in each study were sought (e.g. for all measures, time points, analyses), and if not, the methods used to decide which results to collect.	5
	10b	List and define all other variables for which data were sought (e.g. participant and intervention characteristics, funding sources). Describe any assumptions made about any missing or unclear information.	5
Study risk of bias assessment	11	Specify the methods used to assess risk of bias in the included studies, including details of the tool(s) used, how many reviewers assessed each study and whether they worked independently, and if applicable, details of automation tools used in the process.	5
Effect measures	12	Specify for each outcome the effect measure(s) (e.g. risk ratio, mean difference) used in the synthesis or presentation of results.	6

Reporting bias assessment	14	Describe any methods used to assess risk of bias due to missing results in a synthesis (arising from reporting biases).	7
Certainty assessment	15	Describe any methods used to assess certainty (or confidence) in the body of evidence for an outcome.	7
**RESULTS**			
Study selection	16a	Describe the results of the search and selection process, from the number of records identified in the search to the number of studies included in the review, ideally using a flow diagram.	8, figure 1
	16b	Cite studies that might appear to meet the inclusion criteria, but which were excluded, and explain why they were excluded.	Table S2
Synthesis methods	13a	Describe the processes used to decide which studies were eligible for each synthesis (e.g. tabulating the study intervention characteristics and comparing against the planned groups for each synthesis (item #5)).	6, 7
	13b	Describe any methods required to prepare the data for presentation or synthesis, such as handling of missing summary statistics, or data conversions.	6, 7
	13c	Describe any methods used to tabulate or visually display results of individual studies and syntheses.	6, 7
	13d	Describe any methods used to synthesize results and provide a rationale for the choice(s). If meta-analysis was performed, describe the model(s), method(s) to identify the presence and extent of statistical heterogeneity, and software package(s) used.	6, 7
	13e	Describe any methods used to explore possible causes of heterogeneity among study results (e.g. subgroup analysis, meta-regression).	6, 7
	13f	Describe any sensitivity analyses conducted to assess robustness of the synthesized results.	6, 7
Study characteristics	17	Cite each included study and present its characteristics.	8, table 1
Risk of bias in studies	18	Present assessments of risk of bias for each included study.	Table S3
Results of individual studies	19	For all outcomes, present, for each study: (a) summary statistics for each group (where appropriate) and (b) an effect estimate and its precision (e.g. confidence/credible interval), ideally using structured tables or plots.	9, 10, 11, figure 3, table 2
Results of syntheses	20a	For each synthesis, briefly summarise the characteristics and risk of bias among contributing studies.	8, 9, table 1, table 2
	20b	Present results of all statistical syntheses conducted. If meta-analysis was done, present for each the summary estimate and its precision (e.g. confidence/credible interval) and measures of statistical heterogeneity. If comparing groups, describe the direction of the effect.	9, 10, 11, figure 3, table 2
	20c	Present results of all investigations of possible causes of heterogeneity among study results.	9, 10, 11, figure 4
	20d	Present results of all sensitivity analyses conducted to assess the robustness of the synthesized results.	Figure 4, Figure S19-21
Reporting biases	21	Present assessments of risk of bias due to missing results (arising from reporting biases) for each synthesis assessed.	7

Certainty of evidence	22	Present assessments of certainty (or confidence) in the body of evidence for each outcome assessed.	9, 10, 11, figure 3, table 2
**DISCUSSION**			
Discussion	23a	Provide a general interpretation of the results in the context of other evidence.	11, 12
	23b	Discuss any limitations of the evidence included in the review.	13, 14
	23c	Discuss any limitations of the review processes used.	13, 14
	23d	Discuss implications of the results for practice, policy, and future research.	14
**OTHER INFORMATION**			
Registration and protocol	24a	Provide registration information for the review, including register name and registration number, or state that the review was not registered.	3
	24b	Indicate where the review protocol can be accessed, or state that a protocol was not prepared.	3
	24c	Describe and explain any amendments to information provided at registration or in the protocol.	3
Support	25	Describe sources of financial or non-financial support for the review, and the role of the funders or sponsors in the review.	14
Competing interests	26	Declare any competing interests of review authors.	14
Availability of data, code and other materials	27	Report which of the following are publicly available and where they can be found: template data collection forms; data extracted from included studies; data used for all analyses; analytic code; any other materials used in the review.	14

Search strategy

Final search run on 25/03/2025.

PubMed

("Breast Neoplasms"[Mesh] OR “breast neoplasms” [tiab] OR “breast tumor” [tiab] OR “cancer of breast” [tiab] OR “human mammary carcinomas” [tiab] OR “breast carcinoma” [tiab] OR “breast tumor” [tiab] OR “breast neoplasm” [tiab] OR “breast cancer” [tiab] OR “Neoplasm, Breast” [tiab] OR “Breast Tumors"[tiab] OR “Tumor, Breast” [tiab] OR “Neoplasms, Breast” [tiab] OR “Breast Cancer” [tiab] OR “Cancer, Breast” [tiab] OR “Mammary Cancer” [tiab] OR “Malignant Neoplasm of Breast” [tiab] OR “Breast Malignant Neoplasm” [tiab] OR “Malignant Tumor of Breast” [tiab] OR “Breast Malignant Tumor” [tiab] OR “Cancer of Breast” [tiab] OR “Cancer of the Breast” [tiab] OR “Human Mammary Carcinoma*”[tiab] OR “Human Mammary Carcinoma” [tiab])

AND

("Receptors, CCR7"[Mesh] OR Receptor CCR7 [tiab] OR CCR7 [tiab] OR “CC Chemokine Receptor 7” [tiab] OR CD197 Antigen* [tiab] OR CCR7 Receptor* [tiab] OR CC Chemokine Receptor CCR7[tiab])

Embase

('breast cancer'/exp OR 'breast neoplasms':ti,ab OR 'breast tumor':ti,ab OR 'cancer of breast':ti,ab OR 'human mammary carcinomas':ti,ab OR 'breast carcinoma':ti,ab OR 'breast tumor':ti,ab OR 'breast neoplasm':ti,ab OR 'breast cancer':ti,ab OR 'Neoplasm, Breast':ti,ab OR 'Breast Tumors':ti,ab OR 'Tumor, Breast':ti,ab OR 'Neoplasms, Breast':ti,ab OR 'Breast Cancer':ti,ab OR 'Cancer, Breast':ti,ab OR 'Mammary Cancer':ti,ab OR 'Malignant Neoplasm of Breast':ti,ab OR 'Breast Malignant Neoplasm':ti,ab OR 'Malignant Tumor of Breast':ti,ab OR 'Breast Malignant Tumor':ti,ab OR 'Cancer of Breast':ti,ab OR 'Cancer of the Breast':ti,ab OR 'Human Mammary Carcinoma*':ti,ab OR 'Human Mammary Carcinoma':ti,ab)

AND

('chemokine receptor CCR7'/exp OR 'Receptor CCR7':ti,ab OR CCR7:ti,ab OR 'CC Chemokine Receptor 7':ti,ab OR 'CD197 Antigen*':ti,ab OR 'CCR7 Receptor*':ti,ab OR 'CC Chemokine Receptor CCR7':ti,ab)

Scopus

(INDEXTERMS("Breast Neoplasms") OR TITLE-ABS("breast neoplasms") OR TITLE-ABS("breast tumor") OR TITLE-ABS("cancer of breast") OR TITLE-ABS("human mammary carcinomas") OR TITLE-ABS("breast carcinoma") OR TITLE-ABS("breast tumor") OR TITLE-ABS("breast neoplasm") OR TITLE-ABS("breast cancer") OR TITLE-ABS("Neoplasm, Breast") OR TITLE-ABS("Breast Tumors") OR TITLE-ABS("Tumor, Breast") OR TITLE-ABS("Neoplasms, Breast") OR TITLE-ABS("Breast Cancer") OR TITLE-ABS("Cancer, Breast") OR TITLE-ABS("Mammary Cancer") OR TITLE-ABS("Malignant Neoplasm of Breast") OR TITLE-ABS("Breast Malignant Neoplasm") OR TITLE-ABS("Malignant Tumor of Breast") OR TITLE-ABS("Breast Malignant Tumor") OR TITLE-ABS("Cancer of Breast") OR TITLE-ABS("Cancer of the Breast") OR TITLE-ABS("Human Mammary Carcinoma*") OR TITLE-ABS("Human Mammary Carcinoma"))

AND

(INDEXTERMS("Receptors, CCR7") OR TITLE-ABS("Receptor CCR7") OR TITLE-ABS(CCR7) OR TITLE-ABS("CC Chemokine Receptor 7") OR TITLE-ABS("CD197 Antigen*") OR TITLE-ABS("CCR7 Receptor*") OR TITLE-ABS("CC Chemokine Receptor CCR7"))

Medline EBSCO

((MH "Breast Neoplasms+") OR (TI "breast neoplasms" OR AB "breast neoplasms") OR (TI "breast tumor" OR AB "breast tumor") OR (TI "cancer of breast" OR AB "cancer of breast") OR (TI "human mammary carcinomas" OR AB "human mammary carcinomas") OR (TI "breast carcinoma" OR AB "breast carcinoma") OR (TI "breast tumor" OR AB "breast tumor") OR (TI "breast neoplasm" OR AB "breast neoplasm") OR (TI "breast cancer" OR AB "breast cancer") OR (TI "Neoplasm, Breast" OR AB "Neoplasm, Breast") OR (TI "Breast Tumors" OR AB "Breast Tumors") OR (TI "Tumor, Breast" OR AB "Tumor, Breast") OR (TI "Neoplasms, Breast" OR AB "Neoplasms,

Breast") OR (TI "Breast Cancer" OR AB "Breast Cancer") OR (TI "Cancer, Breast" OR AB "Cancer, Breast") OR (TI "Mammary Cancer" OR AB "Mammary Cancer") OR (TI "Malignant Neoplasm of Breast" OR AB "Malignant Neoplasm of Breast") OR (TI "Breast Malignant Neoplasm" OR AB "Breast Malignant Neoplasm") OR (TI "Malignant Tumor of Breast" OR AB "Malignant Tumor of Breast") OR (TI "Breast Malignant Tumor" OR AB "Breast Malignant Tumor") OR (TI "Cancer of Breast" OR AB "Cancer of Breast") OR (TI "Cancer of the Breast" OR AB "Cancer of the Breast") OR (TI "Human Mammary Carcinoma*" OR AB "Human Mammary Carcinoma*") OR (TI "Human Mammary Carcinoma" OR AB "Human Mammary Carcinoma"))

AND

((MH "Receptors, CCR7+") OR (TI "Receptor CCR7" OR AB "Receptor CCR7") OR (TI CCR7 OR AB CCR7) OR (TI "CC Chemokine Receptor 7" OR AB "CC Chemokine Receptor 7") OR (TI "CD197 Antigen*" OR AB "CD197 Antigen*") OR (TI "CCR7 Receptor*" OR AB "CCR7 Receptor*") OR (TI "CC Chemokine Receptor CCR7" OR AB "CC Chemokine Receptor CCR7"))

Web of Science

(ALL="Breast Neoplasms" OR (TI="breast neoplasms" OR AB="breast neoplasms") OR (TI="breast tumor" OR AB="breast tumor") OR (TI="cancer of breast" OR AB="cancer of breast") OR (TI="human mammary carcinomas" OR AB="human mammary carcinomas") OR (TI="breast carcinoma" OR AB="breast carcinoma") OR (TI="breast tumor" OR AB="breast tumor") OR (TI="breast neoplasm" OR AB="breast neoplasm") OR (TI="breast cancer" OR AB="breast cancer") OR (TI="Neoplasm, Breast" OR AB="Neoplasm, Breast") OR (TI="Breast Tumors" OR AB="Breast Tumors") OR (TI="Tumor, Breast" OR AB="Tumor, Breast") OR (TI="Neoplasms, Breast" OR AB="Neoplasms, Breast") OR (TI="Breast Cancer" OR AB="Breast Cancer") OR (TI="Cancer, Breast" OR AB="Cancer, Breast") OR (TI="Mammary Cancer" OR AB="Mammary Cancer") OR (TI="Malignant Neoplasm of Breast" OR AB="Malignant Neoplasm of Breast") OR (TI="Breast Malignant Neoplasm" OR AB="Breast Malignant Neoplasm") OR (TI="Malignant Tumor of Breast" OR AB="Malignant Tumor of Breast") OR (TI="Breast Malignant Tumor" OR AB="Breast Malignant Tumor") OR (TI="Cancer of Breast" OR AB="Cancer of Breast") OR (TI="Cancer of the Breast" OR AB="Cancer of the Breast") OR (TI="Human Mammary Carcinoma*" OR AB="Human Mammary Carcinoma*") OR (TI="Human Mammary Carcinoma" OR AB="Human Mammary Carcinoma"))

AND

(ALL="Receptors, CCR7" OR (TI="Receptor CCR7" OR AB="Receptor CCR7") OR (TI=CCR7 OR AB=CCR7) OR (TI="CC Chemokine Receptor 7" OR AB="CC Chemokine Receptor 7") OR (TI="CD197 Antigen*" OR AB="CD197 Antigen*") OR (TI="CCR7 Receptor*" OR AB="CCR7 Receptor*") OR (TI="CC Chemokine Receptor CCR7" OR AB="CC Chemokine Receptor CCR7"))

Table S2: Excluded articles at full text screening.

Study	Title	Reason for exclusion
[1]	High expression of the chemokine receptor CCR7 is associated with worse outcome in breast cancer	Wrong Publication Type
[2]	Detecting the role of CCR7-CCL21/CCL19 axis in breast cancer progression and lymph node metastasis incidence	Insufficient Data
[3]	Chemokine receptors in advanced breast cancer: Differential expression in metastatic disease sites with diagnostic and therapeutic implications	Wrong Outcome
[4]	Integrating multi-omics data through deep learning for accurate cancer prognosis prediction	Wrong Outcome
[5]	The chemokine receptor CXCR4 is more frequently expressed in breast compared to other metastatic adenocarcinomas in effusions	Wrong Outcome
[6]	Positive lymph-node breast cancer patients - Activation of NF-κB in tumor-associated leukocytes stimulates cytokine secretion that promotes metastasis via C-C chemokine receptor CCR7	In vitro
[7]	Splicing imbalances in basal-like breast cancer underpin perturbation of cell surface and oncogenic pathways and are associated with patients' survival	Wrong Outcome
[8]	Analysis of breast cancer related gene expression using natural splines and the Cox proportional hazard model to identify prognostic associations	Wrong Outcome
[9]	Combinational polymorphisms of seven CXCL12-related genes are protective against breast cancer in Taiwan	Wrong Outcome
[10]	The relation between ACKR4 and CCR7 genes expression and breast cancer metastasis	Insufficient Data
[11]	Different expression patterns of CXCR4, CCR7, maspin and FOXP3 in luminal breast cancers and their sentinel node metastases	Insufficient Data
[12]	Identification of potential genes correlated with breast cancer metastasis and prognosis	Insufficient Data
[13]	Chemokine axes in breast cancer: Factors of the tumor microenvironment reshape the CCR7-driven metastatic spread of luminal-A breast tumors	Wrong Outcome
[14]	Expression of chemokine receptor CCR7 and its association with lymphatic vessel invasion in breast cancer	Foreign Language
[15]	A novel immune-related prognostic index for predicting breast cancer overall survival	Wrong Outcome
[16]	CCR7 expression in Inflammatory breast cancer	Wrong Publication Type
[17]	C-C Chemokine Receptor 7 in Cancer	Wrong Publication Type
[18]	Implications of inflammatory cell death-related IFNG and co-expressed RNAs (AC006369.1 and CCR7) in breast carcinoma prognosis, and anti-tumor immunity	Wrong Design
[19]	Construction of ceRNA prognostic model based on the CCR7/CCL19 chemokine axis as a biomarker in breast cancer	Wrong Design
[20]	Bioinformatics-based screening and analysis of prognostic genes related to immune infiltration in invasive breast cancer	Foreign Language

Table S3: Quality assessment scores

Study	Format recruitment				Equal retention					Equal ascertainment							Equal implementation						Equal prognosis						Sufficient analysis			Temporal precedence					Sum
	1*	2	3	4	5	6	7	8	9	10	11	12	13	14	15	16	17	18	19	20	21	22	23	24	25	26	27	28	29	30	31	32	33	34	35	36	
[21]	1	1	0	1	1	1	0	0	0	1	1	1	0	0	0	0	0	0	1	1	1	1	0	0	0	0	0	0	1	1	0	1	0	0	0	0	15
[22]	0	1	0	0	1	1	1	0	0	1	1	1	0	0	0	0	0	0	1	1	1	0	0	0	1	0	0	0	1	1	1	0	0	0	0	0	14
[23]	1	1	0	1	1	1	0	0	0	1	1	1	0	0	0	0	0	0	0	1	1	1	0	0	0	0	0	0	1	1	1	0	0	0	0	0	14
[24]	1	1	1	1	1	0	1	0	0	1	1	1	0	0	0	0	0	0	1	1	1	1	0	0	0	0	0	1	1	1	1	0	0	0	0	0	17
[25]	1	1	1	1	1	0	1	0	0	1	1	1	0	0	0	0	0	0	1	1	1	1	0	0	0	0	0	1	1	1	1	0	0	0	0	0	17
[26]	1	1	1	1	1	0	1	0	0	1	1	1	0	0	0	0	0	0	1	1	1	1	0	0	0	0	0	1	1	1	1	1	0	0	0	1	19
[27]	1	1	1	1	1	1	0	0	1	1	1	1	1	0	0	0	0	0	1	1	1	1	0	0	0	0	0	0	1	1	1	1	0	0	0	0	19
[28]	0	1	1	1	1	0	0	0	0	1	1	1	1	1	0	0	0	0	1	1	1	1	0	0	0	0	0	1	1	1	1	1	0	0	0	1	19
[29]	1	1	1	1	1	1	0	0	0	0	1	1	0	0	0	0	0	0	1	1	1	1	0	0	0	0	0	0	0	1	1	0	0	0	1	1	16
[30]	1	1	0	1	1	1	1	0	0	1	1	1	0	0	0	0	0	0	1	1	1	1	0	0	0	0	0	0	1	1	1	1	0	0	0	0	17
[31]	1	1	1	0	1	1	1	0	0	1	1	1	0	0	0	0	0	0	1	1	1	1	0	0	0	0	0	1	1	1	1	1	0	0	0	1	19
[32]	0	1	1	1	1	1	0	0	0	1	1	1	0	0	0	0	0	0	1	1	1	1	0	0	0	0	0	0	1	1	1	0	0	0	0	0	15

***The safeguards are outlined below**

Format recruitment

1. Data collected after the start of the study was not used to exclude participants or to select them into the analysis

2. Participants in all comparison groups met the same eligibility requirements and were from the same population and timeframe

3. Determination of eligibility and assignment to treatment group/ exposure strategy were synchronized

4. None of the eligibility criteria were common effects of exposure and outcome

Equal retention

5. Any attrition (or exclusions after entry) was less than 20% of total participant numbers

6. Missing data was less than 20%

7. Analysis accounted for missing data

8. Exposure variations / treatment deviations were less than 20%

9. Variations in exposure or withdrawals after start of the study were addressed by the analysis

Equal ascertainment

10. Procedures for data collection of covariates were reliable and the same for all participants

11. The outcome was objective and/ or reliably measured

12. Exposures/ interventions were objectively and/ or reliably measured

13. Outcome assessor(s) were blinded

14. Participants were blinded

15. Caregivers were blinded

16. Analyst(s) were blinded

Equal implementation

17. Care was delivered equally to all participants

18. Cointerventions that could impact the outcome were comparable between groups or avoided

19. Control and active interventions/ exposures were sufficiently distinct

20. Exposure/intervention definition was consistently applied to all participants

21. Outcome definition was consistently applied to all participants

22. The time period between exposure and outcome was similar across patients and between groups or the analyses adjusted for different lengths of follow-up of patients

Equal prognosis

23. Design and/or analysis strategies were in place that addressed potential confounding

24. Key confounders addressed through design or analysis were not common effects of exposure and outcome

25. Key baseline characteristics / prognostic indicators for the study were comparable across groups

26. Participants were randomly allocated to groups with an adequate randomization process

27. Allocation procedure was adequately concealed

28. Conflict of interests were declared and absent

Sufficient analysis

29. Analytic method was justified by study design or data requirements

30. Computation errors or contradictions were absent

31. There was no discernible data dredging or selective reporting of the outcomes

Temporal precedence

32. All subjects were selected prior to intervention/ exposure and evaluated prospectively

33. Carry-over or refractory effects were avoided or considered in the design of the study or were not relevant

34. The intervention/ exposure period was long enough to have influenced the study outcome

35. Dose of intervention/ exposure was sufficient to influence the outcome

36. Length of follow-up was not too long or too short in relation to the outcome assessment

Figure S1: Funnel plot for age publication bias

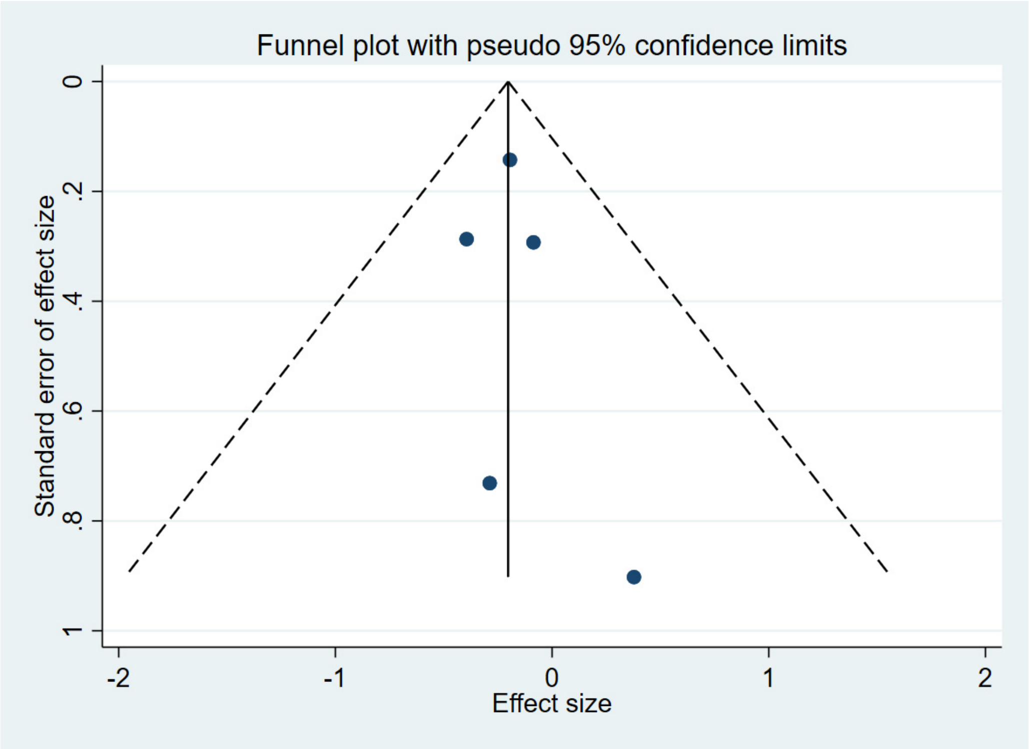

Figure S2: Doi plot for age publication bias

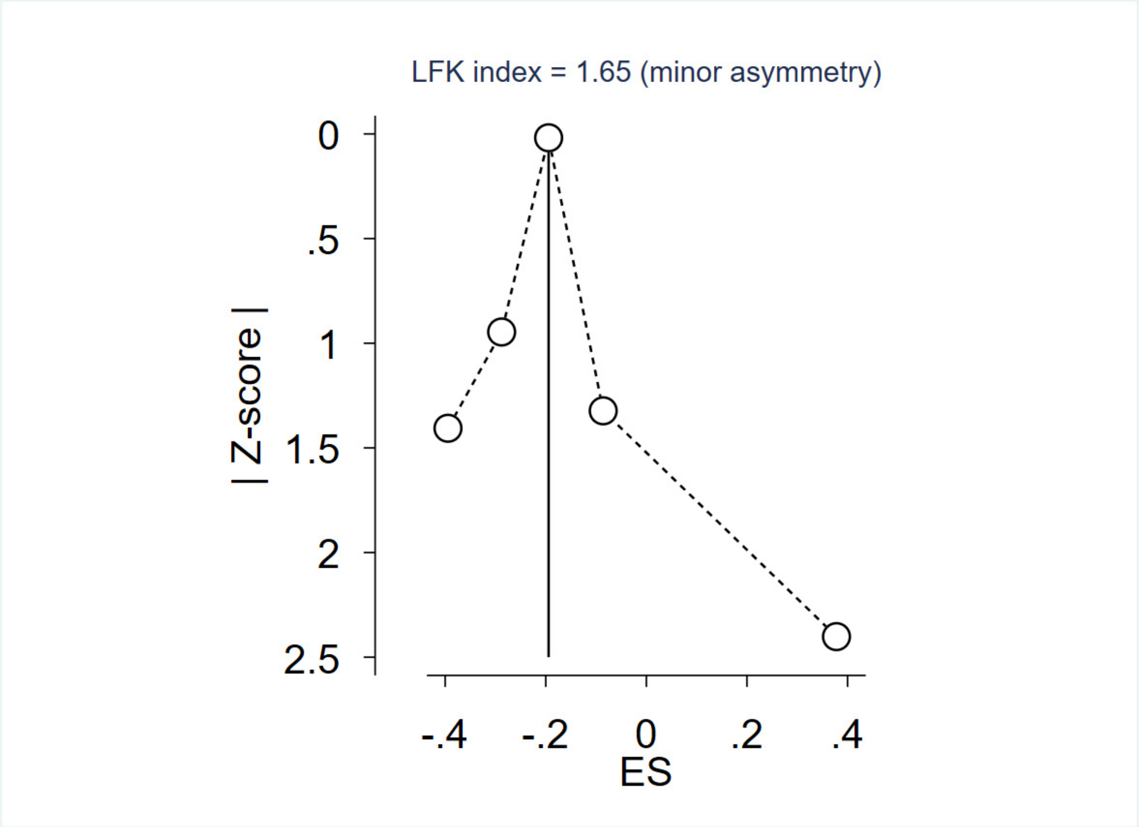

Figure S3: Funnel plot for tumor size publication bias

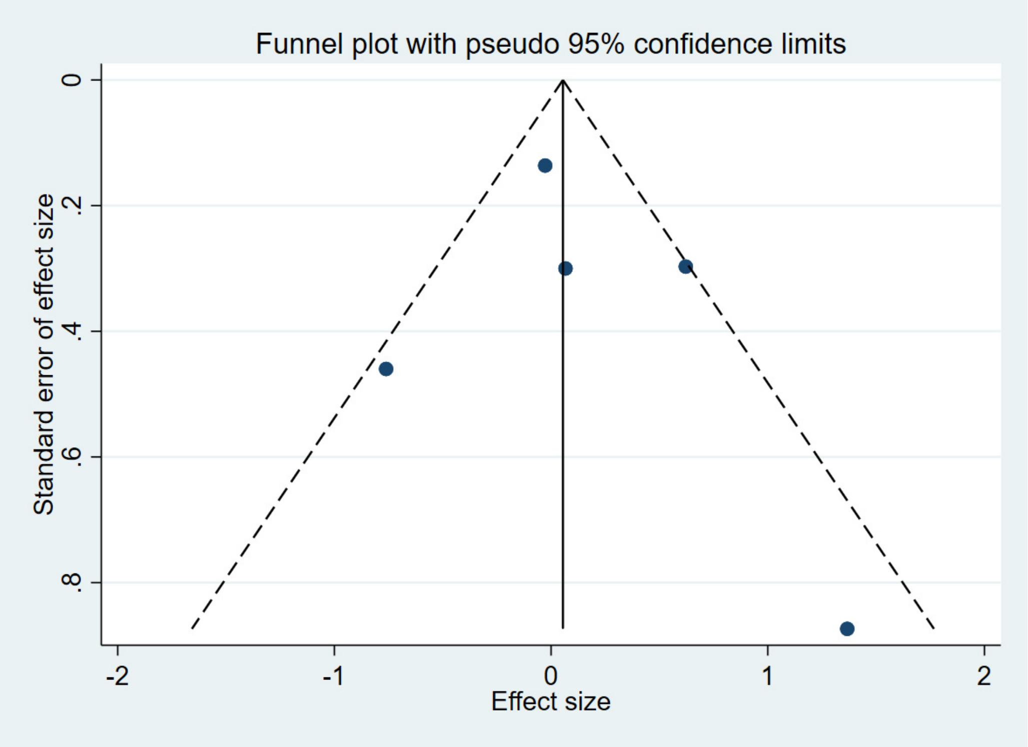

Figure S4: Doi plot for tumor size publication bias

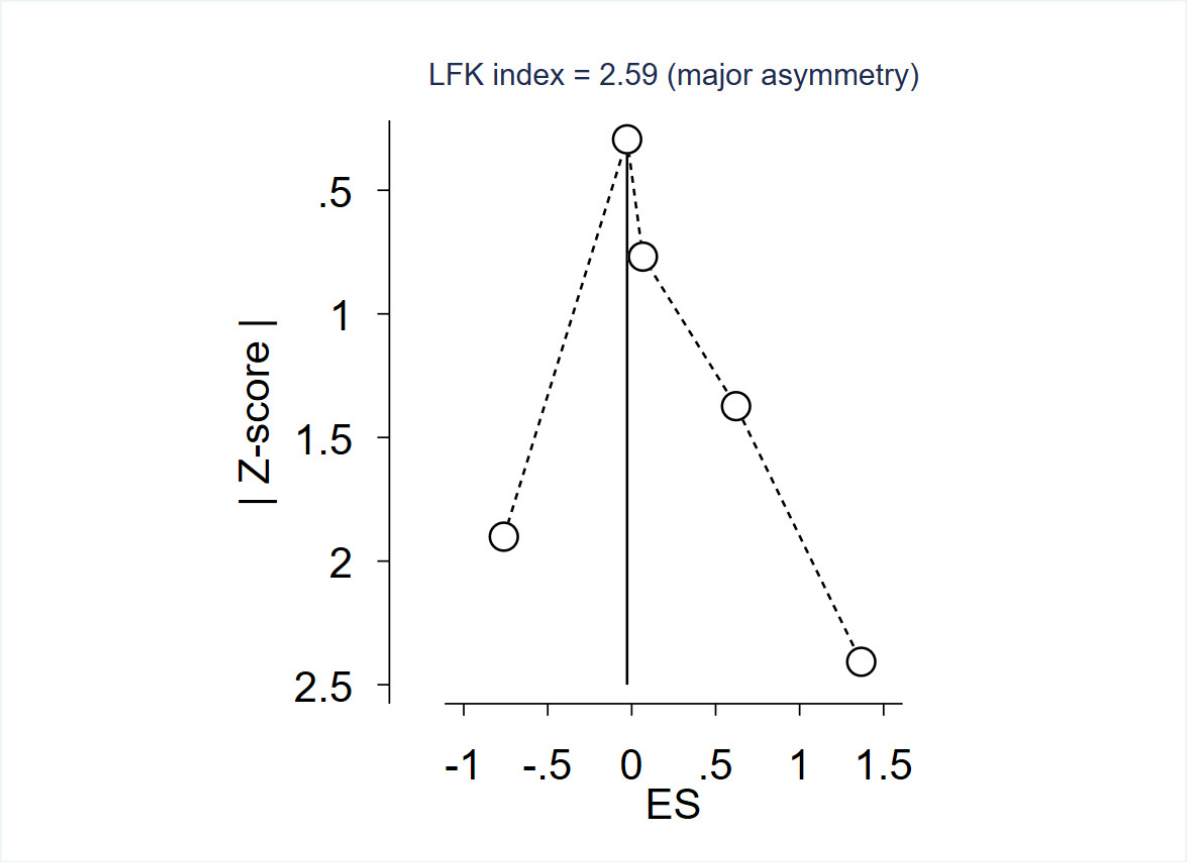

Figure S5: Funnel plot for clinical stage publication bias

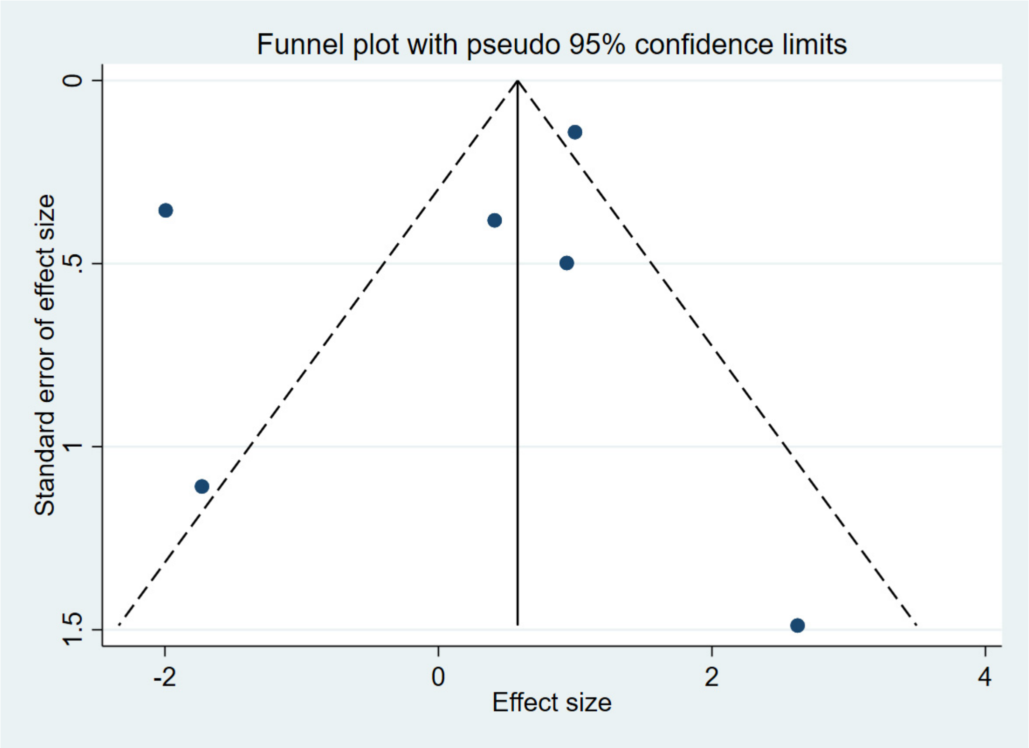

Figure S6: Doi plot for clinical stage publication bias

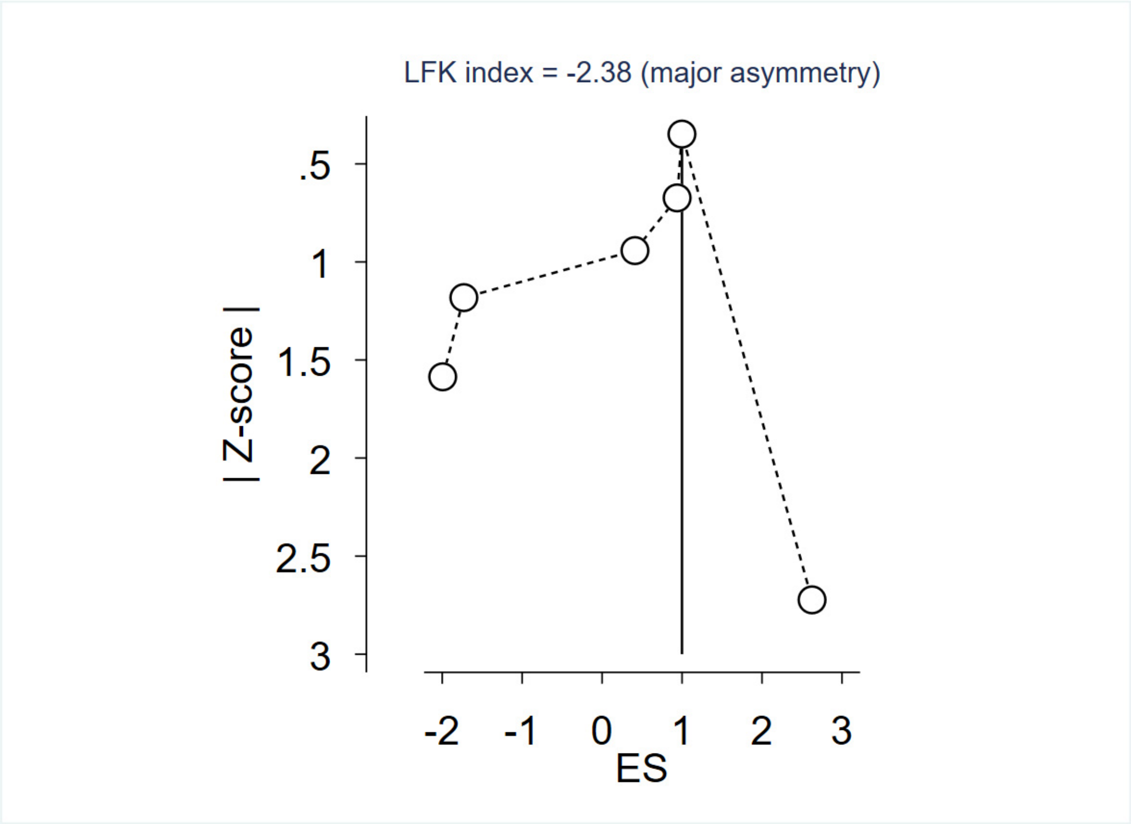

Figure S7: Funnel plot for nodal metastasis publication bias

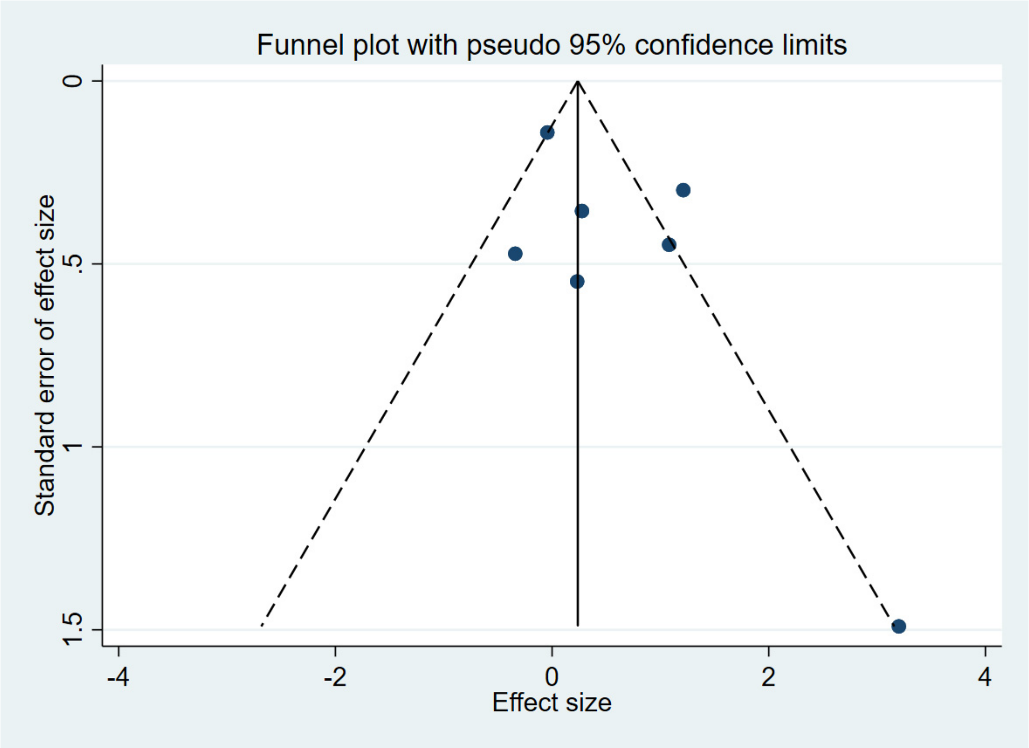

Figure S8: Doi plot for nodal metastasis publication bias

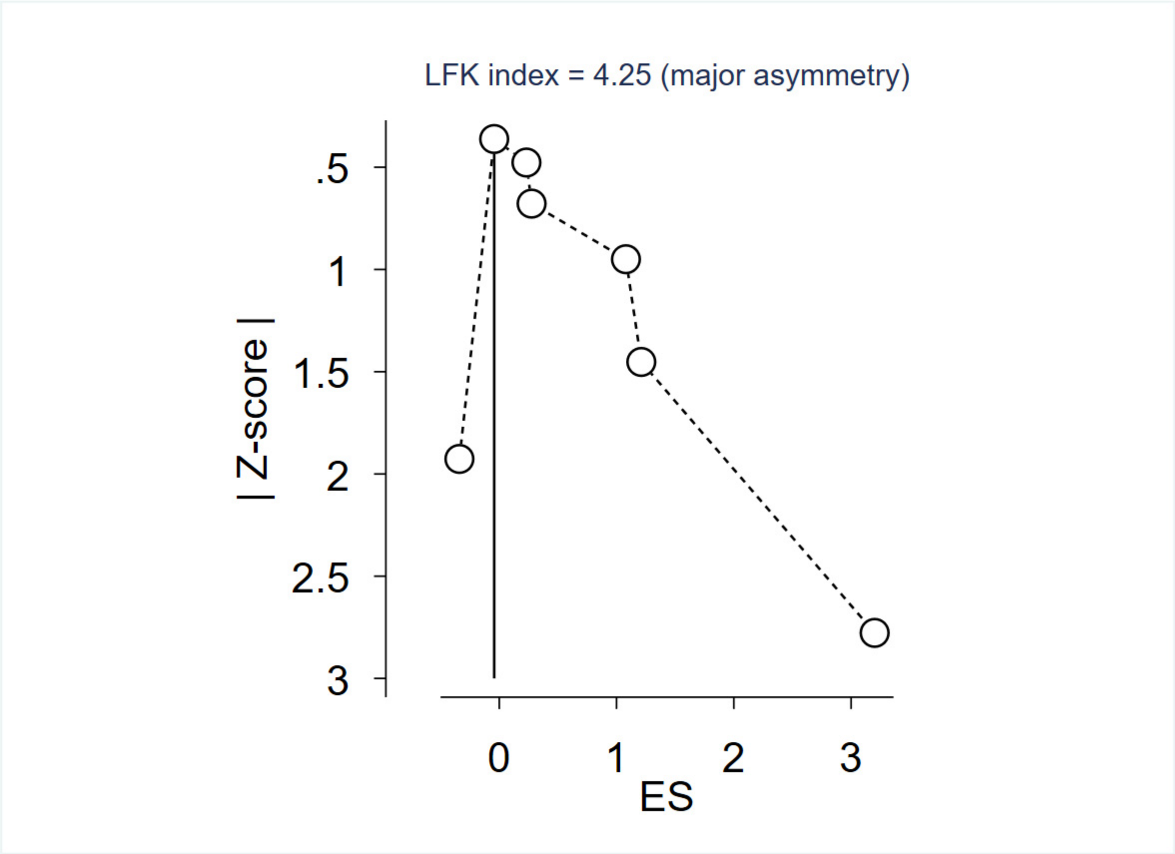

Figure S9: Funnel plot for histological differentiation publication bias

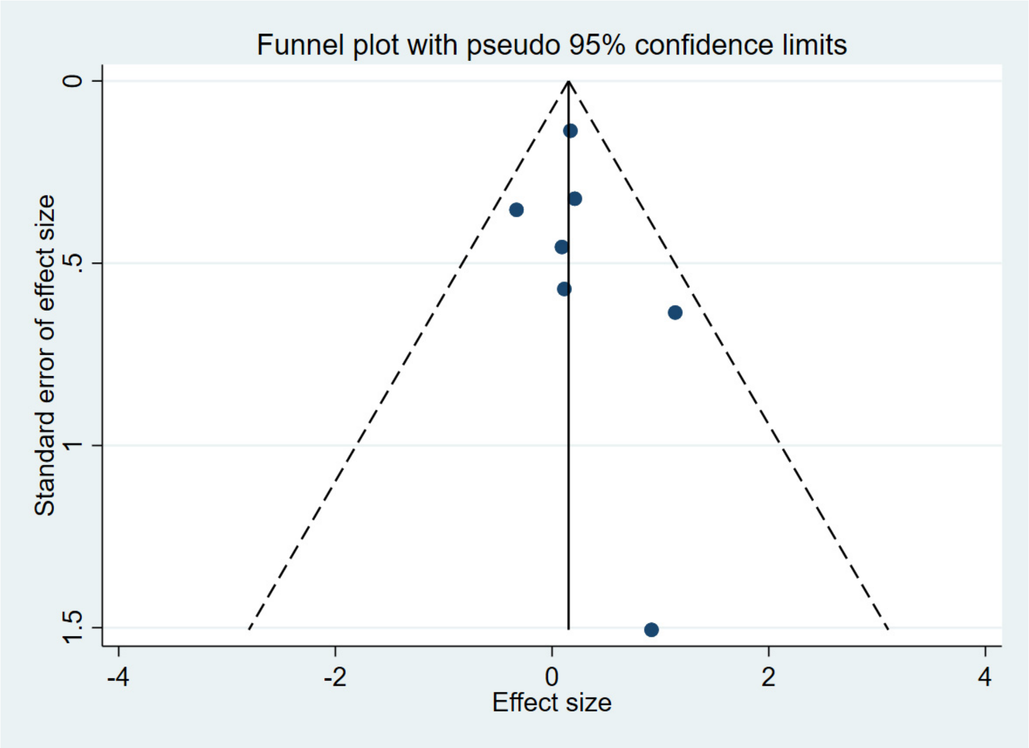

Figure S10: Doi plot for histological differentiation publication bias

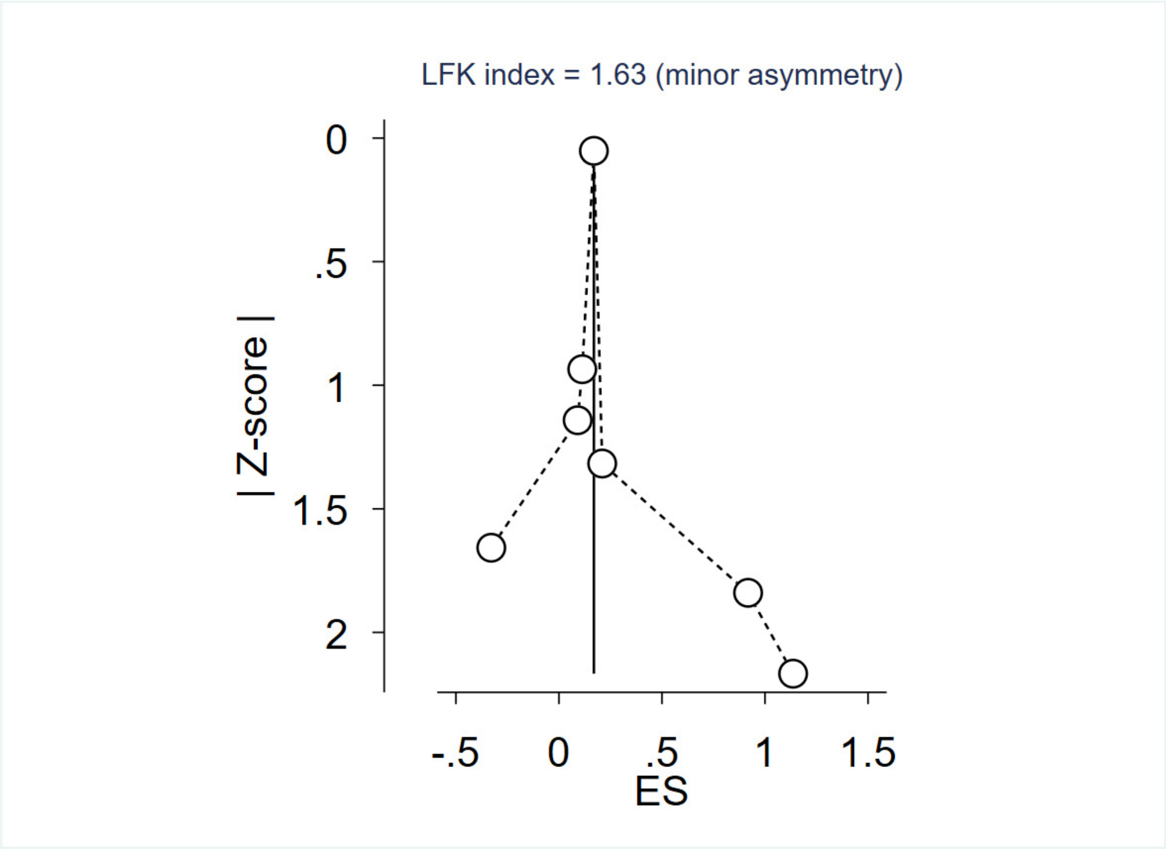

Figure S11: Funnel plot for estrogen receptor publication bias

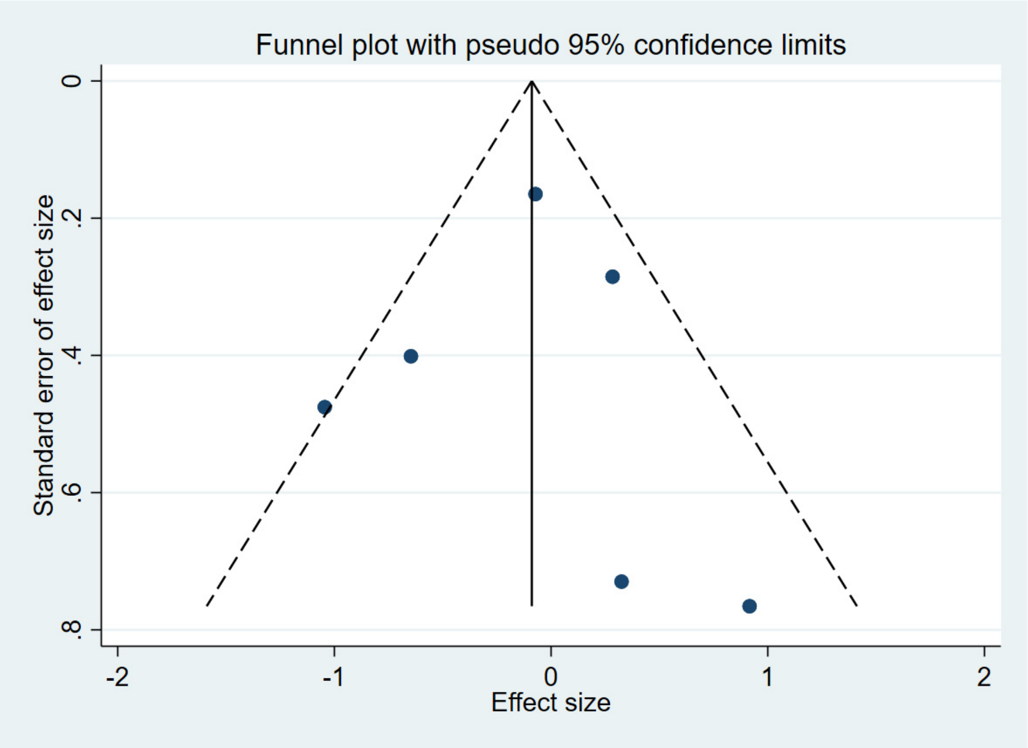

Figure S12: Doi plot for estrogen receptor publication bias

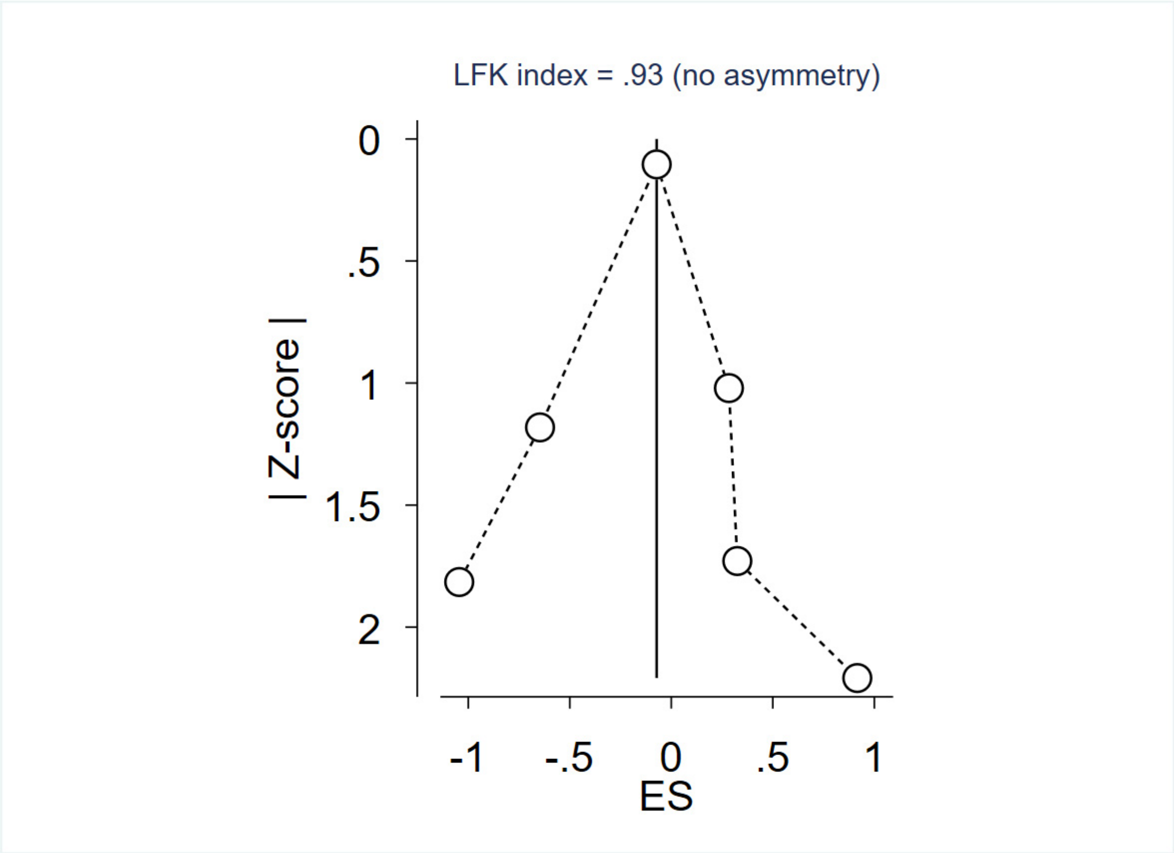

Figure S13: Funnel plot for progesterone receptor publication bias

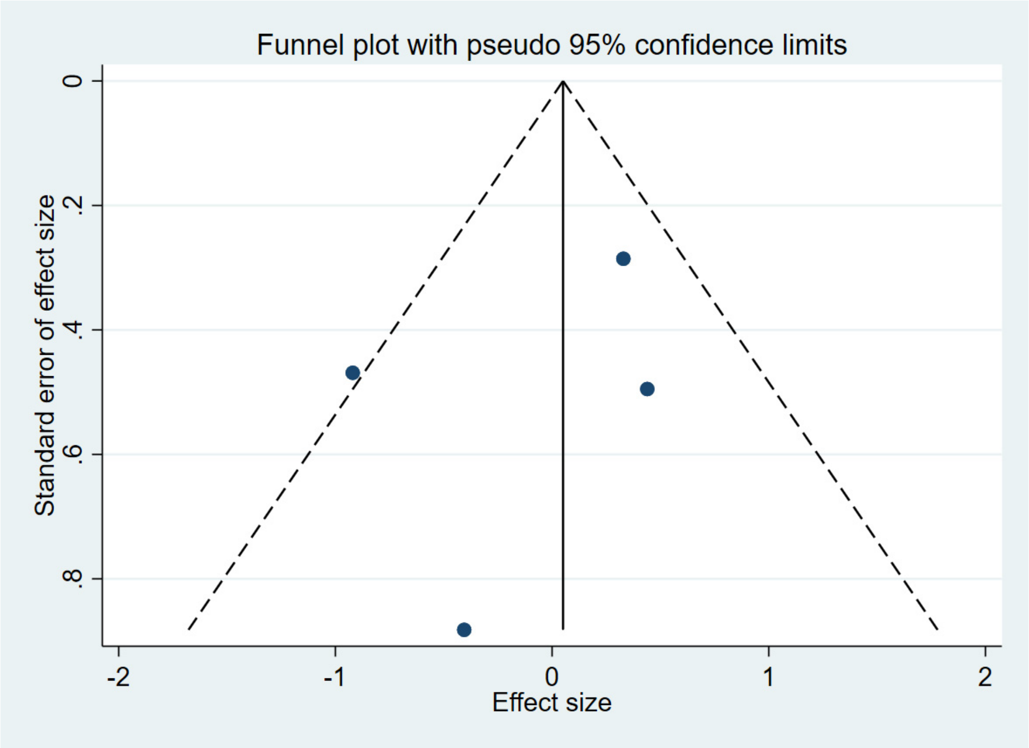

Figure S14: Doi plot for progesterone receptor publication bias

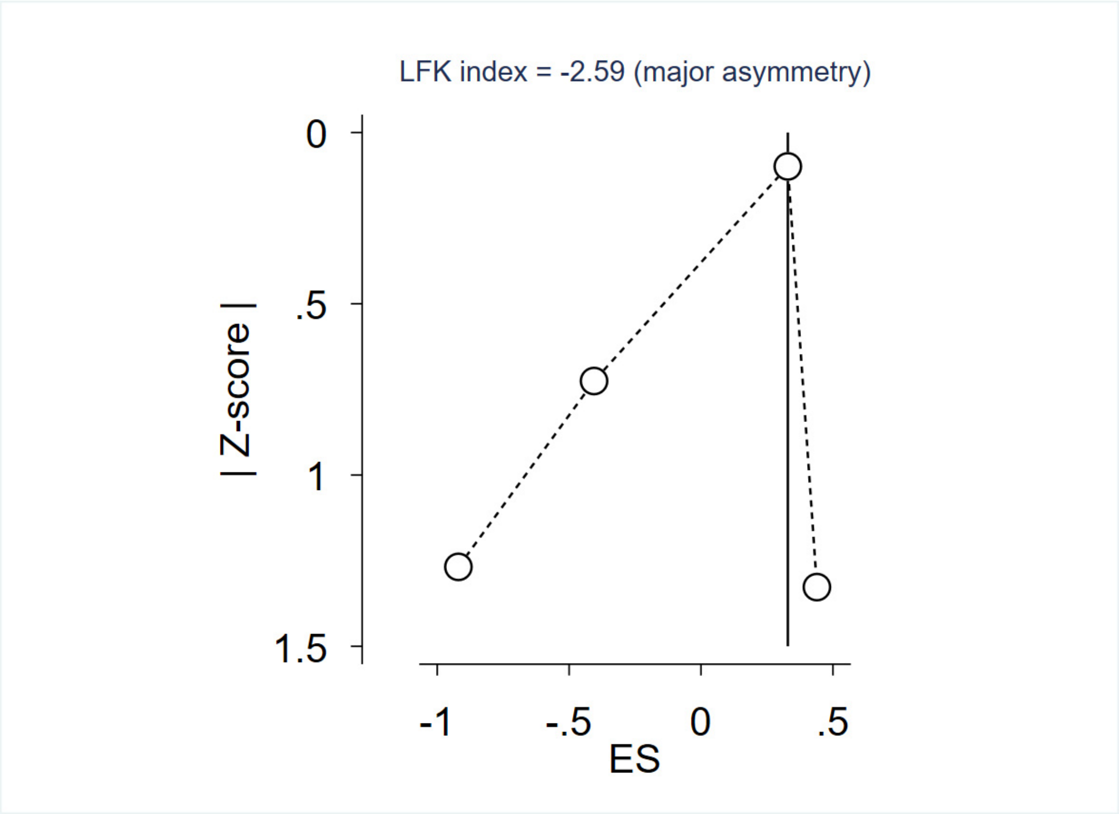

Figure S15: Funnel plot for HER2/neu publication bias

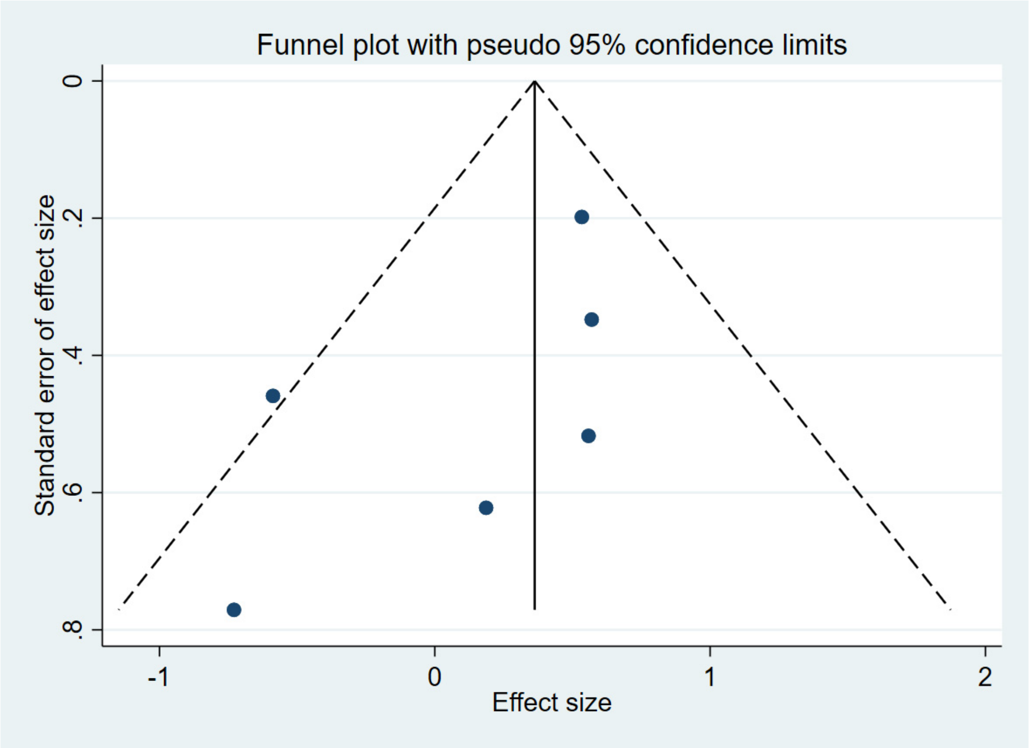

Figure S16: Doi plot for HER2/neu publication bias

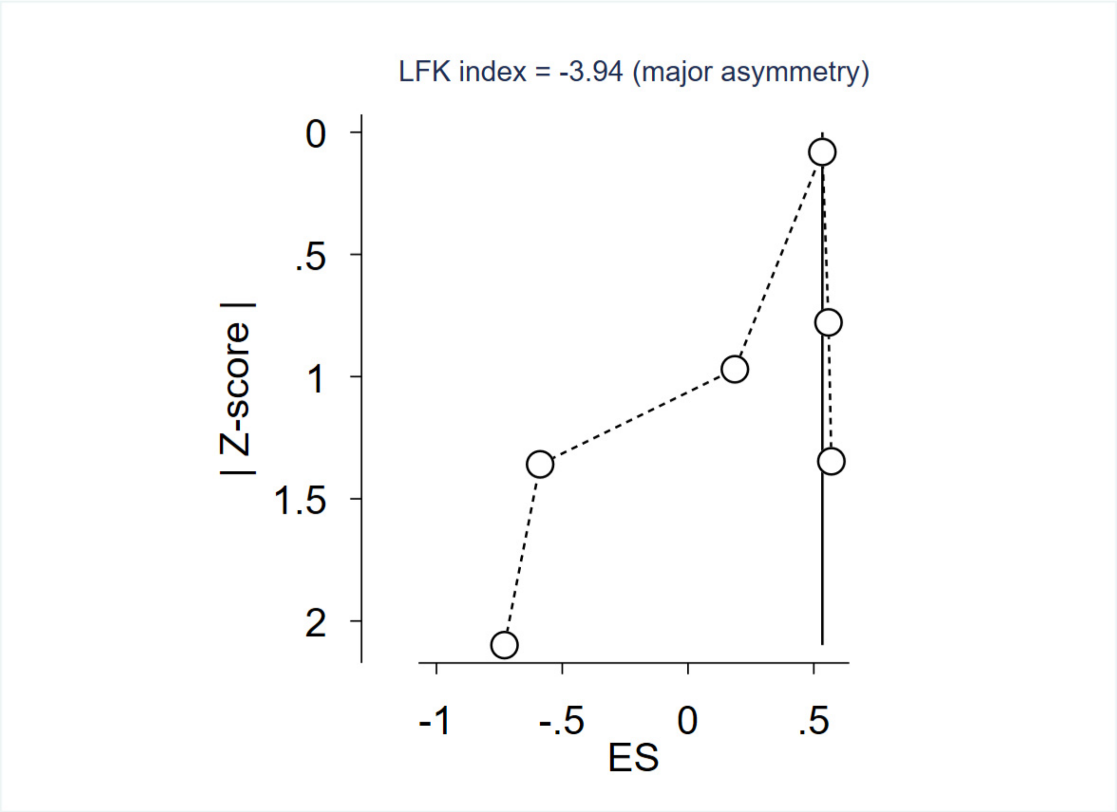

Figure S17: Funnel plot for overall survival publication bias

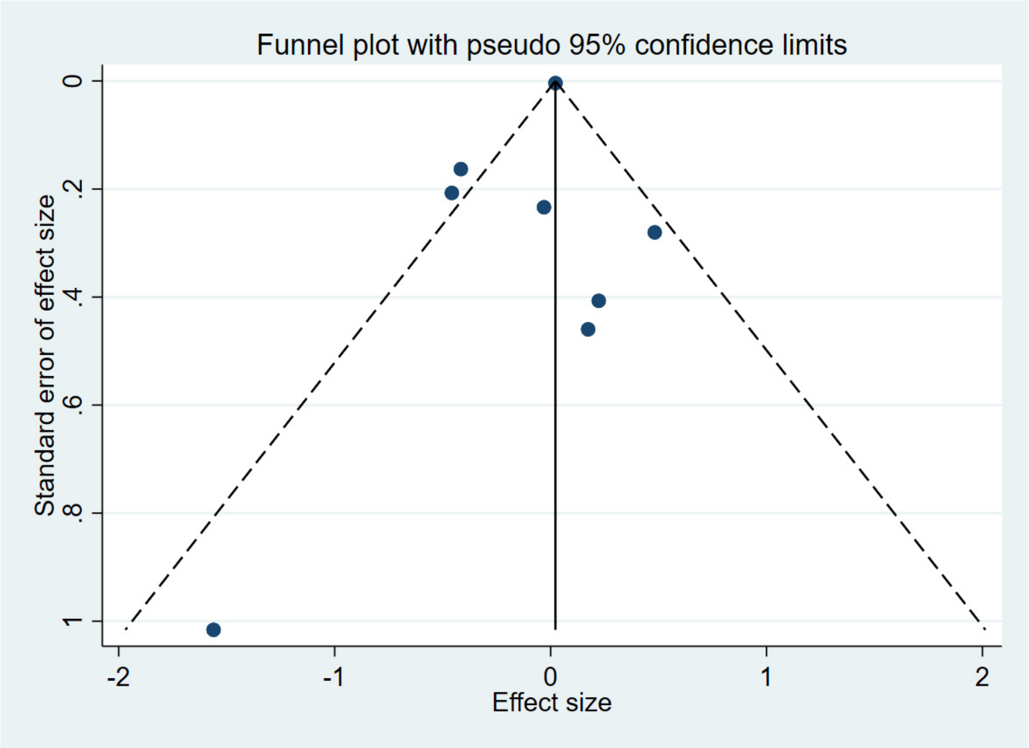

Figure S18: Doi plot for overall survival publication bias

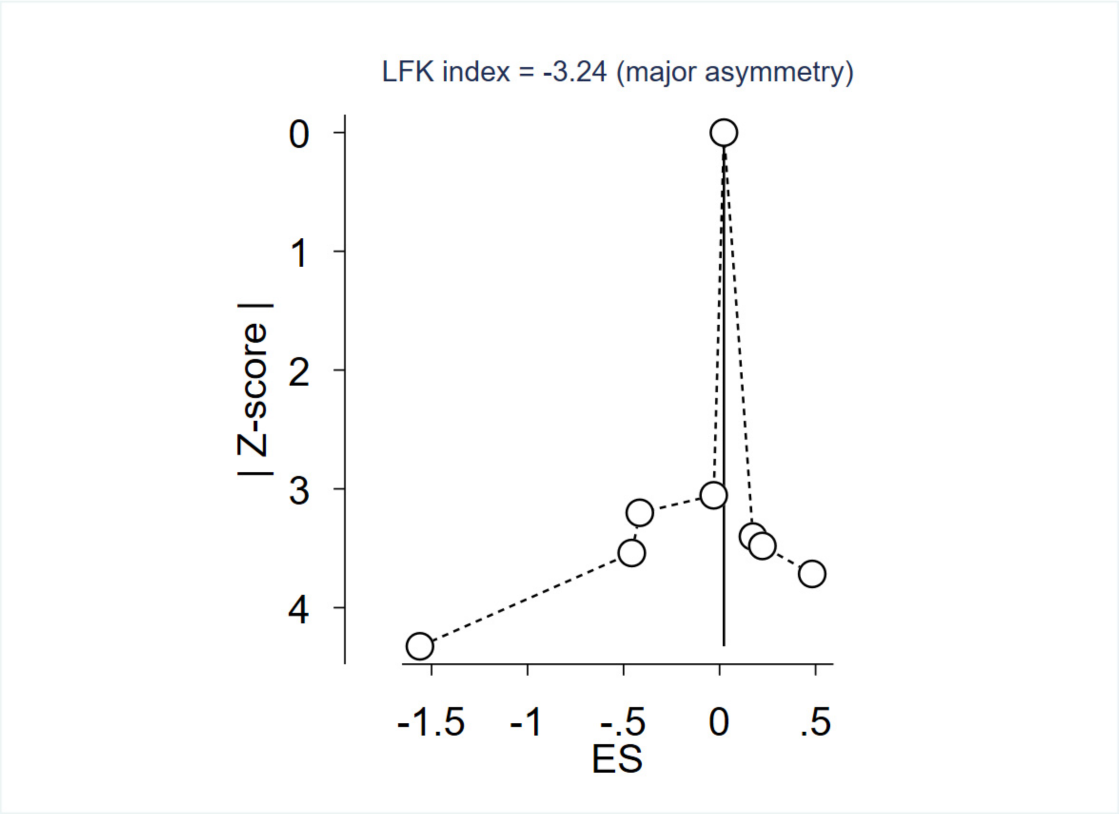

Figure S19: Leave-one-out analysis for tumor size

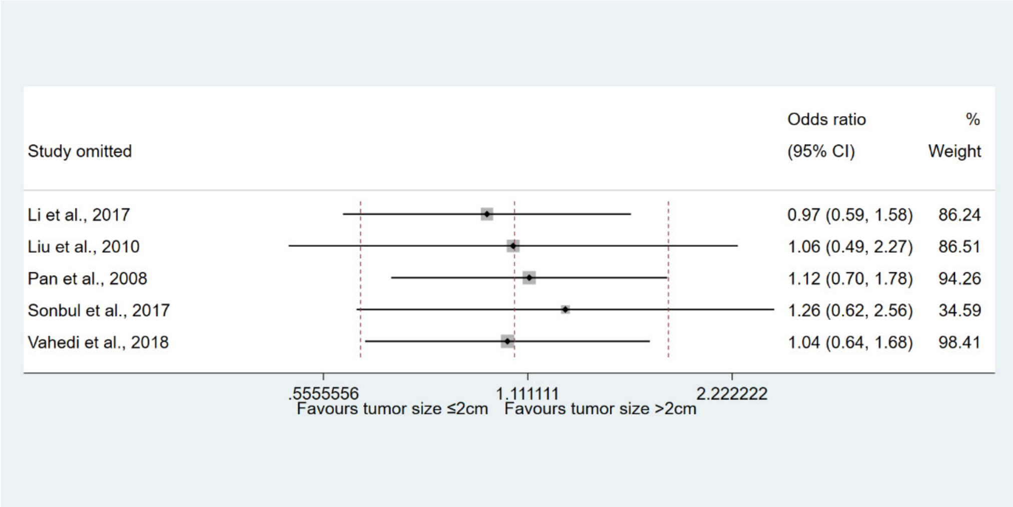

Figure S20: Leave-one-out analysis for estrogen receptor

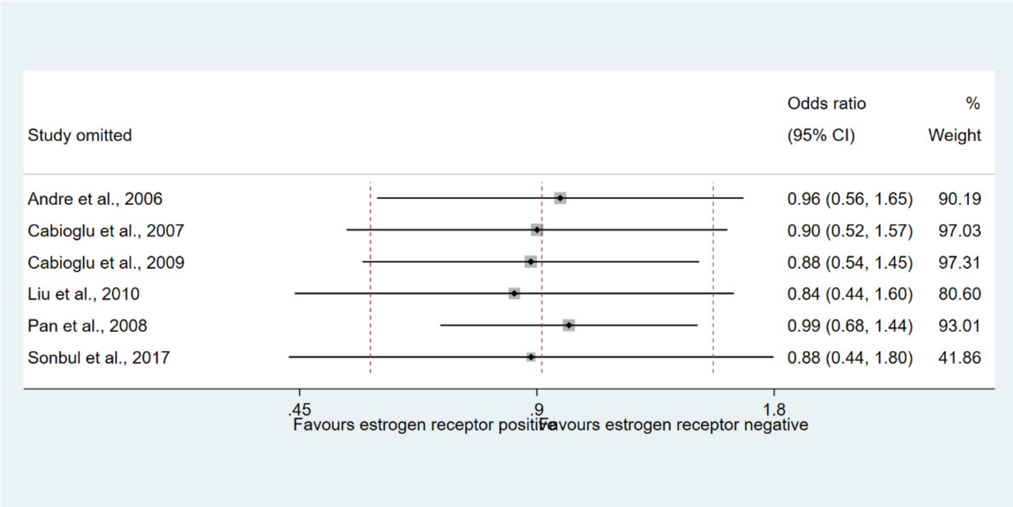

Figure S21: Leave-one-out analysis for progesterone receptor

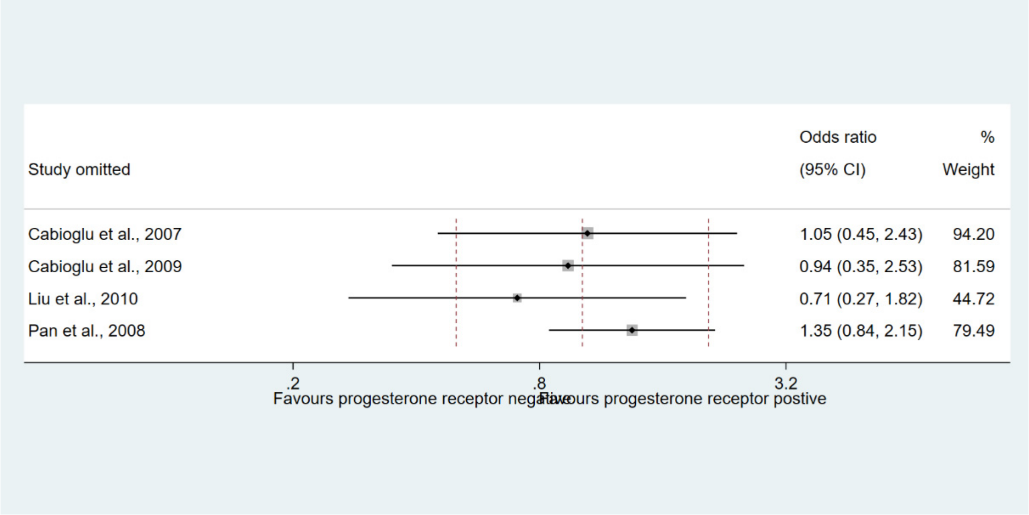

Reference list

[1] M.M. Abu-Khalaf, J. Wheler, M. Zerkowski, R.L. Camp, D.L. Rimm, G.G. Chung, High expression of the chemokine receptor CCR7 is associated with worse outcome in breast cancer, 23(16) (2005) 859S-859S.
[2] K. Aloss, S. Jdeed, Z. Alshehabi, Detecting the role of CCR7-CCL21/CCL19 axis in breast cancer progression and lymph node metastasis incidence, 11(1) (2018) 231-235.
[3] N. Cabioglu, A.A. Sahin, P. Morandi, F. Meric-Bernstam, R. Islam, H.Y. Lin, C.D. Bucana, A.M. Gonzalez-Angulo, G.N. Hortobagyi, M. Cristofanilli, Chemokine receptors in advanced breast cancer: Differential expression in metastatic disease sites with diagnostic and therapeutic implications, 20(6) (2009) 1013-1019.
[4] H. Chai, X. Zhou, Z. Zhang, J. Rao, H. Zhao, Y. Yang, Integrating multi-omics data through deep learning for accurate cancer prognosis prediction, 134 (2021).
[5] B. Davidson, H.P. Dong, A. Holth, A. Berner, B. Risberg, The chemokine receptor CXCR4 is more frequently expressed in breast compared to other metastatic adenocarcinomas in effusions, 14(5) (2008) 476-482.
[6] E.A. El-Ghonaimy, M. El-Shinawi, S.A. Ibrahim, H. El-Ghazaly, R. Abd-El-Tawab, M.A. Nouh, T. El-Mamlouk, M.M. Mohamed, Positive lymph-node breast cancer patients - Activation of NF-κB in tumor-associated leukocytes stimulates cytokine secretion that promotes metastasis via C-C chemokine receptor CCR7, 282(2) (2015) 271-282.
[7] F. Gracio, B. Burford, P. Gazinska, A. Mera, A. Mohd Noor, P. Marra, C. Gillett, A. Grigoriadis, S. Pinder, A. Tutt, E. de Rinaldis, Splicing imbalances in basal-like breast cancer underpin perturbation of cell surface and oncogenic pathways and are associated with patients' survival, 7 (2017) 40177.
[8] B. Kreike, G. Hart, H. Bartelink, M.J. Van De Vijver, Analysis of breast cancer related gene expression using natural splines and the Cox proportional hazard model to identify prognostic associations, 122(3) (2010) 711-720.
[9] G.T. Lin, H.F. Tseng, C.H. Yang, M.F. Hou, L.Y. Chuang, H.T. Tai, M.H. Tai, Y.H. Cheng, C.H. Wen, C.S. Liu, C.J. Huang, C.L. Wang, H.W. Chang, Combinational polymorphisms of seven CXCL12-related genes are protective against breast cancer in Taiwan, 13(2) (2009) 165-172.
[10] M.M. Mohammed, O. Shaker, M.M. Ramzy, S.S. Gaber, H.S. Kamel, M.F. Abed El Baky, The relation between ACKR4 and CCR7 genes expression and breast cancer metastasis, 279 (2021).
[11] L. Strien, K. Joensuu, P. Heikkilä, M.H. Leidenius, Different expression patterns of CXCR4, CCR7, maspin and FOXP3 in luminal breast cancers and their sentinel node metastases, 37(1) (2017) 175-182.
[12] C. Tan, F. Zuo, M.Q. Lu, S. Chen, Z.Z. Tian, Y. Hu, Identification of potential genes correlated with breast cancer metastasis and prognosis, 15(1) (2022) 126-133.
[13] P. Weitzenfeld, O. Kossover, C. Körner, T. Meshel, S. Wiemann, D. Seliktar, D.F. Legler, A. Ben-Baruch, Chemokine axes in breast cancer: Factors of the tumor microenvironment reshape the CCR7-driven metastatic spread of luminal-A breast tumors, 99(6) (2016) 1009-1025.
[14] J. Xie, Y. Zhou, L.J. Fan, Q.Q. Chen, J. Jiang, Expression of chemokine receptor CCR7 and its association with lymphatic vessel invasion in breast cancer, 15(6) (2008) 437-439.
[15] X. Yu, J. Guo, Q. Zhou, W. Huang, C. Xu, X. Long, A novel immune-related prognostic index for predicting breast cancer overall survival, 28(2) (2021) 434-447.
[16] W. Balema, S. Krishnamurthy, A. Lawrence, M. Rodriguez, R. Larson, N. Fowlkes, N.T. Ueno, W. Woodward, Abstract P2-26-15: CCR7 expression in Inflammatory breast cancer, Cancer Research 83(5_Supplement) (2023) P2-26-15-P2-26-15.
[17] C.A. Bill, C.M. Allen, C.M. Vines, C-C Chemokine Receptor 7 in Cancer, Cells 11(4) (2022) 656.
[18] Y. Deng, Z. Li, M. Pan, H. Wu, B. Ni, X. Han, Implications of inflammatory cell death-related IFNG and co-expressed RNAs (AC006369.1 and CCR7) in breast carcinoma prognosis, and anti-tumor immunity, Frontiers in Genetics Volume 14 - 2023 (2023).

[19] R. Ma, X. Guan, N. Teng, Y. Du, S. Ou, X. Li, Construction of ceRNA prognostic model based on the CCR7/CCL19 chemokine axis as a biomarker in breast cancer, BMC Medical Genomics 16(1) (2023) 254.
[20] 王明霞, 马学琳, 黑君虎, 杨轩, 王娅娜, 基于生物信息学筛选并分析与浸润性乳腺癌免疫浸润有关的预后基因, 中国免疫学杂志 39(9) (2023) 1878-1884,1892.
[21] F. Andre, N. Cabioglu, H. Assi, J.C. Sabourin, S. Delaloge, A. Sahin, K. Broglio, J.P. Spano, C. Combadiere, C. Bucana, J.C. Soria, M. Cristofanilli, Expression of chemokine receptors predicts the site of metastatic relapse in patients with axillary node positive primary breast cancer, Annals of Oncology 17(6) (2006) 945-951.
[22] N. Cabioglu, Y. Gong, R. Islam, K.R. Broglio, N. Sneige, A. Sahin, A.M. Gonzalez-Angulo, P. Morandi, C. Bucana, G.N. Hortobagyi, M. Cristofanilli, Expression of growth factor and chemokine receptors: New insights in the biology of inflammatory breast cancer, Annals of Oncology 18(6) (2007) 1021-1029.
[23] N. Cabioglu, M.S. Yazici, B. Arun, K.R. Broglio, G.N. Hortobagyi, J.E. Price, A. Sahin, CCR7 and CXCR4 as novel biomarkers predicting axillary lymph node metastasis in T1 breast cancer, Clinical Cancer Research 11(16) (2005) 5686-5693.
[24] D. Chen, C. Bao, F. Zhao, H. Yu, G. Zhong, L. Xu, S. Yan, Exploring Specific miRNA-mRNA Axes With Relationship to Taxanes-Resistance in Breast Cancer, Frontiers in Oncology 10 (2020).
[25] X.W. Du, G. Li, J. Liu, C.Y. Zhang, Q. Liu, H. Wang, T.S. Chen, Comprehensive analysis of the cancer driver genes in breast cancer demonstrates their roles in cancer prognosis and tumor microenvironment, World Journal of Surgical Oncology 19(1) (2021).
[26] D.C. Gurgel, D.V.T. Wong, A.M. Bandeira, J.F.B. Pereira, J.V. Gomes-Filho, A.C. Pereira, P.G. Barros Silva, F.R.F. Távora, A.F. Pereira, R.C.P. Lima-Júnior, P.R.C. Almeida, Cytoplasmic CCR7 (CCR7c) immunoexpression is associated with local tumor recurrence in triple-negative breast cancer, Pathology Research and Practice 216(12) (2020).
[27] P.J. Lamy, F. Fina, C. Bascoul-Mollevi, A.C. Laberenne, P.M. Martin, L. Ouafik, W. Jacot, Quantification and clinical relevance of gene amplification at chromosome 17q12-q21 in human epidermal growth factor receptor 2-amplified breast cancers, Breast Cancer Research 13(1) (2011).
[28] X. Li, S. Sun, N. Li, J. Gao, J. Yu, J. Zhao, M. Li, Z. Zhao, High Expression of CCR7 Predicts Lymph Node Metastasis and Good Prognosis in Triple Negative Breast Cancer, Cellular Physiology and Biochemistry 43(2) (2017) 531-539.
[29] Y. Liu, R. Ji, J. Li, Q. Gu, X. Zhao, T. Sun, J. Wang, J. Li, Q. Du, B. Sun, Correlation effect of EGFR and CXCR4 and CCR7 chemokine receptors in predicting breast cancer metastasis and prognosis, Journal of Experimental and Clinical Cancer Research 29(1) (2010).
[30] M.R. Pan, M.F. Hou, H.C. Chang, W.C. Hung, Cyclooxygenase-2 up-regulates CCR7 via EP2/EP4 receptor signaling pathways to enhance lymphatic invasion of breast cancer cells, Journal of Biological Chemistry 283(17) (2008) 11155-11163.
[31] S.N. Sonbul, K.L. Gorringe, M.A. Aleskandarany, A. Mukherjee, A.R. Green, I.O. Ellis, E.A. Rakha, Chemokine (C-C motif) receptor 7 (CCR7) associates with the tumour immune microenvironment but not progression in invasive breast carcinoma, Journal of Pathology: Clinical Research 3(2) (2017) 105-114.
[32] L. Vahedi, M. Ghasemi, J. Yazdani, S. Ranjbar, B. Nouri, A. Alizadeh, P. Afshar, Investigation of CCR7 marker expression using immunohistochemical method and its association with clinicopathologic properties in patients with breast cancer, International Journal of Hematology-Oncology and Stem Cell Research 12(2) (2018) 103-110.
